# Dynamic caveolae exclude bulk membrane proteins and are required for sorting of excess glycosphingolipids

**DOI:** 10.1038/ncomms7867

**Published:** 2015-04-21

**Authors:** Elena Shvets, Vassilis Bitsikas, Gillian Howard, Carsten Gram Hansen, Benjamin J. Nichols

**Affiliations:** 1 MRC-LMB, Francis Crick Avenue, Cambridge CB2 0QH, UK; 2 Sanford Consortium for Regenerative Medicine UCSD, 2880 Torrey Pines Scenic Drive, La Jolla, California 92037, USA

## Abstract

Caveolae have long been implicated in endocytosis. Recent data question this link, and in the absence of specific cargoes the potential cellular function of caveolar endocytosis remains unclear. Here we develop new tools, including doubly genome-edited cell lines, to assay the subcellular dynamics of caveolae using tagged proteins expressed at endogenous levels. We find that around 5% of the cellular pool of caveolae is present on dynamic endosomes, and is delivered to endosomes in a clathrin-independent manner. Furthermore, we show that caveolae are indeed likely to bud directly from the plasma membrane. Using a genetically encoded tag for electron microscopy and ratiometric light microscopy, we go on to show that bulk membrane proteins are depleted within caveolae. Although caveolae are likely to account for only a small proportion of total endocytosis, cells lacking caveolae show fundamentally altered patterns of membrane traffic when loaded with excess glycosphingolipid. Altogether, these observations support the hypothesis that caveolar endocytosis is specialized for transport of membrane lipid.

## Abstract (web summary)

Recent data question the long-assumed link between caveolae and endocytosis of membrane proteins. Shvets *et al*. use genome editing to tag and trace endogenous caveolar proteins, providing evidence that these structures exclude membrane proteins and are instead required for proper sorting of excess membrane lipids.

## Graphical abstract

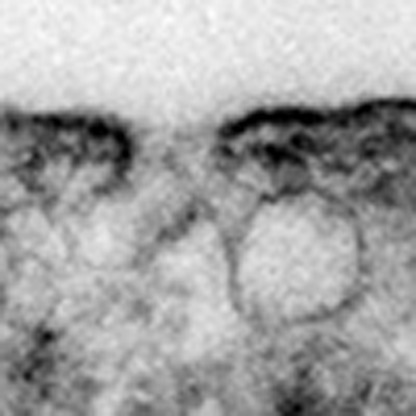

Caveolae are flask-shaped invaginations of the plasma membrane found in vertebrate cells. Despite the fact that caveolae were first identified by electron microscopy over 50 years ago, their functions are still not fully understood. They are thought to have roles in protecting cells against damage from mechanical stress, in a range of signalling pathways, in vesicle trafficking, and in lipid homeostasis^1^,^2^,^3^.

Recently, there has been considerable progress in understanding how caveolae are generated. The membrane bulb of caveolae is shaped by a caveolar coat complex, composed of caveolin and cavin proteins^4^,^5^. Caveolins are membrane proteins embedded in the inner leaflet of the plasma membrane, while cavins are soluble proteins recruited to caveolin oligomers in the plasma membrane to form the coat complex^6^,^7^,^8^,^9^,^10^,^11^. The ATPase EHD2, which regulates caveolar dynamics, has a separate distribution at the neck of caveolae, and additional proteins including pacsin 2 and dynamin 2 may also be present at the neck^4^,^12^,^13^,^14^,^15^,^16^,^17^,^18^. Both caveolin 1 and cavin 1 are essential components of caveolae^19^,^20^,^21^.

In mammals, caveolae are likely to protect the plasma membrane from physical damage^22^,^23^. They can disassemble under mechanical stress, and may thereby act as a reservoir of membrane to allow cells to stretch^23^. This could explain some of the phenotypes of vertebrates without caveolae, for example, muscular dystrophy^19^,^24^. In this context, the phylogenetic distribution of caveolins and cavins is intriguing. Caveolins are found in invertebrate species including some insects and nematodes (http://www.treefam.org/family/TF315736), while cavins are apparently restricted to vertebrates (http://www.treefam.org/family/TF331031). This suggests that there is an ancestral function for caveolin, and that additional functions may have evolved along with the cavin proteins. In *caveolin* mutant nematodes there are changes in lipid distribution in intestinal epithelial cells, leading to the hypothesis that the ancestral caveolin function could be related to lipid transport^25^. One of the most striking phenotypes of humans and mice lacking caveolae is lipodystrophy^26^. Humans without functional *cavin 1* completely lack subcutaneous fat^24^,^27^, while *cavin 1* knockout mice have a more mild lipodystrophy and complex metabolic changes^19^,^28^. *Caveolin 1* knockout mice are also lipodystrophic, are resistant to diet-induced obesity and display altered metabolism consistent with adipocyte dysfunction^26^,^29^,^30^.

One outstanding and central issue pertaining to the cell biological function of caveolae is the extent to which they are involved in intracellular trafficking, potentially by acting as autonomous vesicles. Recent developments question this idea. First, evidence that the virus SV40 provides a marker for caveolar endocytosis^31^,^32^,^33^,^34^ has been disputed by more recent data^35^,^36^, and the literature does not reveal further good candidates for endocytic cargoes that are specific to caveolae. Second, it is now clear that overexpression of caveolin-1-GFP causes aberrant accumulation in endosomal compartments that may be mistaken for caveolae-positive endosomes^36^,^37^. Previous data had reported that overexpressed caveolin-1-GFP can readily be observed in motile intracellular structures^38^, but whether these are induced by overexpression and whether they contain endocytosed cargo of some kind remains to be addressed. Third, both caveolin and cavin proteins are required to produce a functional caveolar coat and hence caveolar morphology^4^,^9^,^10^,^39^. Therefore, data on the dynamics of caveolin 1 alone^31^,^38^,^40^, without additional information as to whether cavin proteins are present in the same vesicle, does not amount to evidence for trafficking of intact caveolae. Finally, experiments using biotinylation of the total complement of plasma membrane proteins reveal that most endocytosis takes place via clathrin-coated pits. Co-localization between endocytosed proteins and caveolin 1, though detected, is minimal^41^.

A second important set of open questions relate to how the caveolar coat influences the local composition of the plasma membrane within caveolae. Despite considerable effort, specific protein cargoes for caveolae have yet to be conclusively identified in non-specialised cell types, hampering study of caveolae budding from the plasma membrane and contributing to the lack of clarity as to their role in trafficking^1^. Literature invoking the presence of specific caveolar binding domains in cell signalling receptors and other proteins has been refuted by structural analysis^42^, and the extent to which generic membrane proteins can freely diffuse into the caveolar membrane has yet to be investigated. Several reports associate caveolae with trafficking of glycosphingolipids, but whether this is then necessarily coupled to transport of membrane proteins in the same region of the plasma membrane is not clear^26^,^43^.

One further area of uncertainty, clearly linked to the questions of the endocytic activity and membrane content of caveolae outlined above, relates to the cellular function of caveolar dynamics. Although changes in cellular lipid composition in response to deletion of caveolar genes have been reported^44^, the extent to which the pattern of intracellular membrane traffic of different lipid species is altered without caveolae requires further investigation^43^,^45^. Lipid-binding bacterial toxin subunits such as the B-subunits of shiga and cholera toxins are thought to enter the cell via multiple endocytic pathways^46^,^47^, and in the absence of evidence for highly specific sorting of lipids into caveolae or other plasma membrane structures it is likely that this is also the case for endogenous lipid species.

In this study we used genome editing technology to develop tools to address the three principal issues outlined above; the extent to which caveolae are involved directly in endocytosis, the composition of the caveolar membrane and hence likely function of this endocytosis, and the extent to which patterns of cellular membrane traffic are altered without caveolae. Imaging data demonstrate that caveolae are highly dynamic, and do indeed act as endocytic vesicles. However, quantitative analysis of endocytic flux through caveolae shows that they account for only a small fraction of total endocytosis, and both ratiometric light microscopy and electron microscopy demonstrate that bulk plasma membrane proteins are excluded from caveolae. In the light of this, and of a third tranche of new experimental data showing that changes in cellular membrane trafficking in response to loading of excess glycosphingolipid are dependent on caveolae, we propose that one cellular function of caveolar endocytosis is to maintain plasma membrane lipid homeostasis.

## Results

### Genome-edited caveolin-1-GFP and cavin-1-mCherry

**Figure 1:**
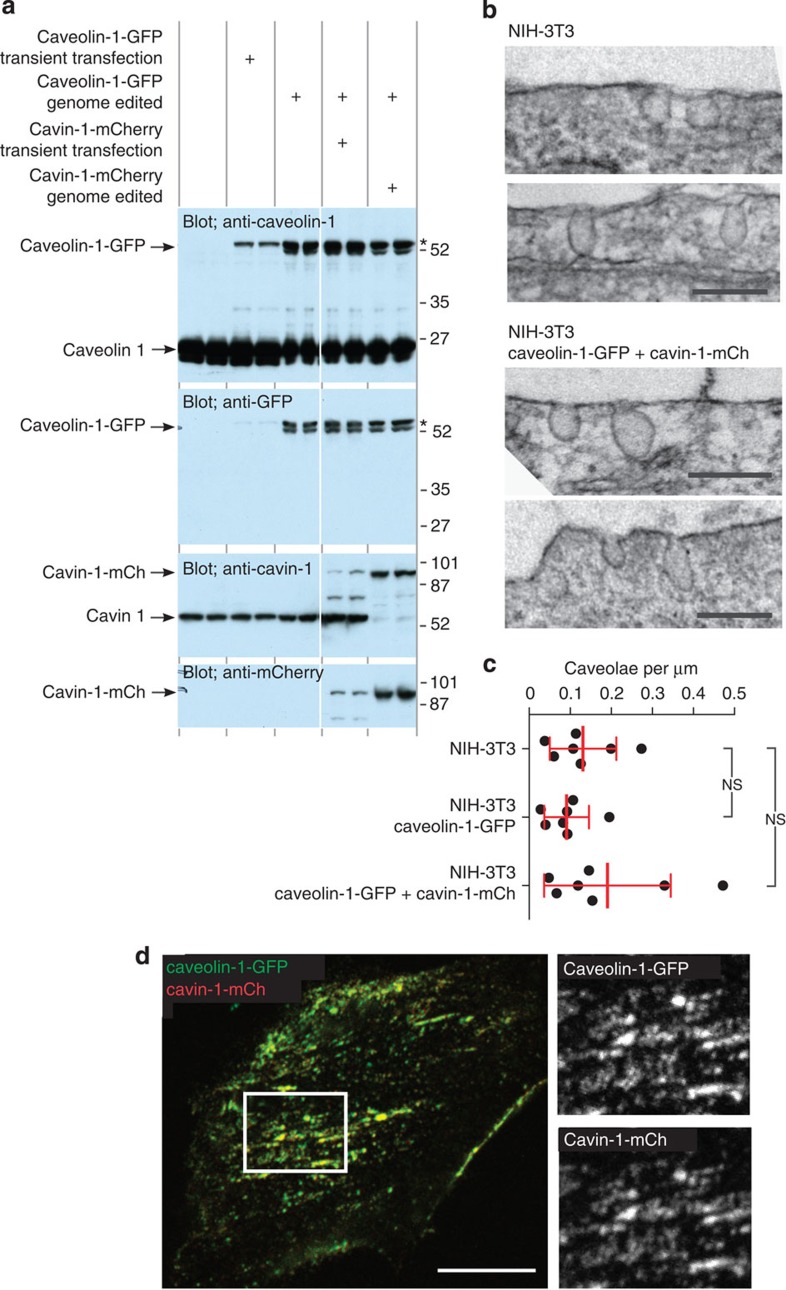
Genome editing to produce caveolin-1-GFP and cavin-1-mCherry does not perturb caveolar morphology or abundance. (**a**) NIH3T3 cells untransfected, overexpressing either caveolin-1-GFP or cavin-1-mCherry via CMV-promoter plasmid and transient transfection, or expressing either caveolin-1-GFP or both caveolin-1-GFP and cavin-1-mCherry from endogenous loci via genome editing to insert GFP or mCherry, were analysed by western blotting using the antibodies as shown. All the samples were obtained and analysed in duplicate. *Note that caveolin-1-GFP expressed from endogenous loci appears in two isoforms, similarly to endogenous untagged protein (visible at shorter exposures). (**b**) Electron micrographs of wild-type NIH3T3 cells or genome-edited NIH3T3 cells expressing both caveolin-1-GFP and cavin-1-mCherry. Bars are 100 nm. (**c**) Quantification of the abundance of caveolae in genome-edited cell lines used in this study. Each point represents one cell perimeter outlined in 10–30 individual electron micrographs. Bars are mean and s.d. (**d**) Confocal image of live NIH3T3 cells expressing both caveolin-1-GFP and cavin-1-mCherry from endogenous loci. Bar is 10 μm, the white box delineates the region shown at higher magnification in the right-hand panels.

We used a site-specific TALE nuclease and gene targeting by homologous recombination to produce an NIH3T3 cell line expressing caveolin-1-GFP by insertion of GFP coding sequence at the 3' end of the protein coding sequence of the endogenous *caveolin 1* gene^48^. Western blotting suggested that one allele only is targeted, as endogenous caveolin 1 is also expressed in this cell line (Fig. 1a). The caveolin-1-GFP cell line was then subjected to a second round of gene targeting, using cas9 RNA-directed nuclease^49^ and homologous recombination to insert mCherry coding sequence at the 3′ end of the protein coding sequence of the endogenous *cavin 1* gene (formal gene name is PTRF^50^). Evidently both *cavin 1* alleles were targeted as the band corresponding to endogenous cavin 1 disappeared in Western blots of the cell line, and was replaced by a band the size of cavin-1-mCherry (Fig. 1a). Electron microscopy was used to confirm that expression of caveolin-1-GFP and cavin-1-mCherry from endogenous genomic loci does not alter the abundance or morphology of caveolae (Fig. 1b,c).

Overexpression of caveolin-1-GFP results in rapid turnover via ubiquitination and endocytosis, and accumulation of overexpressed caveolin 1 in endosomes^36^. We confirmed that endogenously expressed caveolin-1-GFP is as stable as endogenous caveolin 1, and is much more stable than when overexpressed (Supplementary Fig. 1). Endogenously expressed caveolin-1-GFP is therefore unlikely to be mis-localized to endosomes as a result of increased turnover^36^. Confocal microscopy was used to examine the distribution of caveolin-1-GFP and cavin-1-mCherry in the genome-edited NIH3T3 cell line. There was remarkably complete co-localization between the two proteins (Fig. 1d). Unless otherwise explicitly stated, all further experiments in this paper use caveolin-1-GFP and cavin-1-mCherry expressed in the genome-edited cell lines.

### Caveolae are present in dynamic intracellular structures

**Figure 2:**
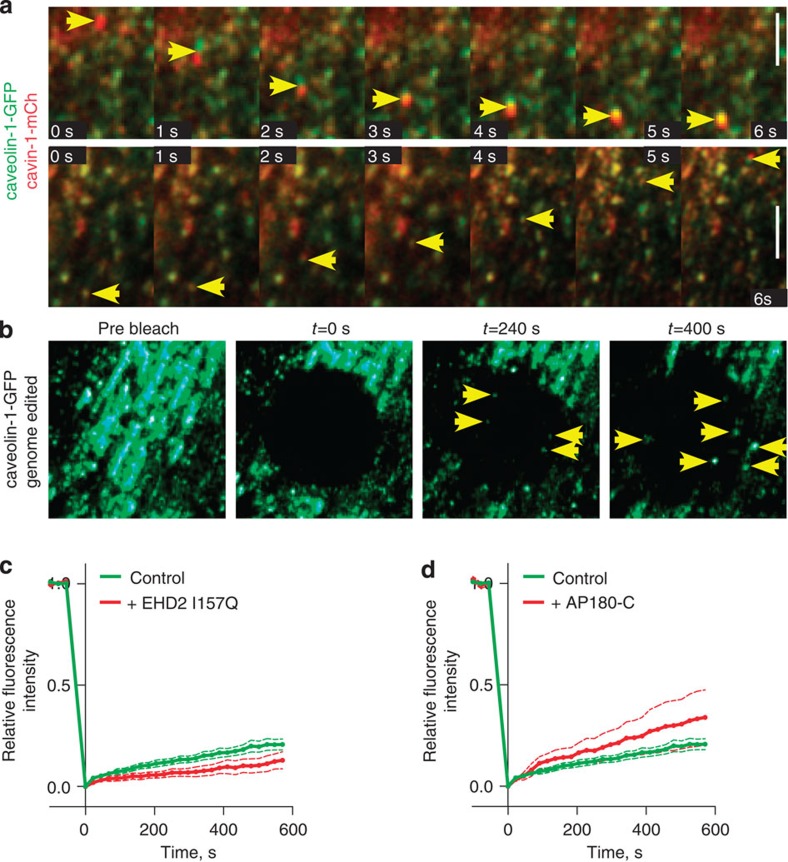
Genome-edited caveolin-1-GFP and cavin-1-mCherry are present in dynamic intracellular vesicles. (**a**) Time-lapse TIR imaging of live NIH3T3 cells expressing both genome-edited caveolin-1-GFP and cavin-1-mCherry shows that these proteins are present on motile structures. Bars are 2 μm. Note that in the upper series of images the motile structure is brighter than minimum quantal caveolin-1-GFP+cavin-1-mCherry puncta visible in the same images, while in the lower series the motile structure is more likely to represent one quantal assembly of caveolin-1-GFP+cavin-1-mCherry. See also Supplementary Movies 2 and 3. (**b**) Time-lapse confocal imaging of a typical FRAP experiment in genome-edited cells expressing caveolin-1-GFP shows fluorescence recovery due to movement of vesicular structures, rather than association of new molecules to existing structures in the bleached area. See also Supplementary Movie 4. (**c**) FRAP experiments comparing the mobility of genome-edited caveolin-1-GFP in untransfected cells to cells expressing EHD2-I157Q-mCherry. *N*=58 for control, 17 for mutant, bars are 95% confidence intervals. (**d**) FRAP experiments comparing the mobility of genome-edited caveolin-1-GFP in untransfected cells to cells expressing AP180-C-myc. *N*=58 for control, 19 for mutant, bars are 95% confidence intervals. The control data are the same as in **c**.

Time-lapse imaging revealed two characteristic behaviours for structures containing caveolin-1-GFP and cavin-1-mCherry. They were either static or motile (Fig. 2a, Supplementary Movie 1). The striking and readily visualized motile structures were both small puncta likely to represent individual caveolae, and larger structures that may represent quantal assemblies of multiple caveolae in one organelle^51^ (Fig. 2a, best seen in Supplementary Movie 1, structures in Fig. 2a are shown in Supplementary Movies 2 and 3). Both cavin 1 and caveolin 1 were present in the motile structures at the same relative intensity to that observed in static plasma membrane structures, suggesting that they contain single or multiple caveolae by the criterion of possessing an assembled caveolar coat. In contrast to caveolin-1-GFP expressed at endogenous levels in the genome-edited cells, overexpressed caveolin-1-GFP was readily detected in structures that lack cavin 1 and are, therefore, unlikely to represent caveolae (Supplementary Fig. 2).

Fluorescence recovery after photobleaching (FRAP) was used to quantify the proportion of caveolin-1-GFP in mobile structures. After 10 min of recovery, around 10% of the original caveolin-1-GFP fluorescence had recovered (Fig. 2b,c). Importantly, this recovery was clearly due to movement of vesicular structures (Fig. 2b, Supplementary Movie 4). Overexpression of caveolin-1-GFP significantly increased the proportion of caveolin-1-GFP structures that are motile (Supplementary Fig. 3).

The ATPase EHD2 has been implicated in regulating the budding of caveolae from the plasma membrane^12^,^13^,^16^,^17^. To ascertain whether the motile fraction of caveolae detected in FRAP experiments is contingent on EHD2-regulated, clathrin-independent budding from the plasma membrane we carried out FRAP in cells overexpressing either the I157Q mutant of EHD2, which has increased ATPase activity and stabilises caveolae at the plasma membrane^12^,^13^, or the C-terminal clathrin-binding domain of AP180, which interferes with formation of clathrin-coated pits^52^. EHD2 I157Q reduced the mobility of caveolae as previously reported in experiments following overexpressed caveolin-1-GFP (Fig. 2c)^12^,^13^. In contrast, AP180-C significantly increased the mobility of caveolae (Fig. 2d). The effect of the EHD2 157Q mutant suggests that caveolar motility measured by FRAP is indeed dependent on the stability of caveolae at the plasma membrane. These data suggest that caveolar dynamics are not contingent on the activity of clathrin-coated pits.

### Caveolae are present in endosomes

The dynamic and highly motile nature of 5–10% of caveolar structures containing caveolin-1-GFP and cavin-1-mCherry suggested that they represent intracellular vesicular organelles, as rapid movements in and out of the field of total internal reflection (TIR) illumination, as well as lateral movements at speeds of up to 0.5 μms^−1^, are inconsistent with the relevant structure being part of the continuous plasma membrane (Fig. 2a, Supplementary Movies 1,2,3,4). We sought additional evidence to address the question of whether caveolae are present in endocytic vesicles.

**Figure 3:**
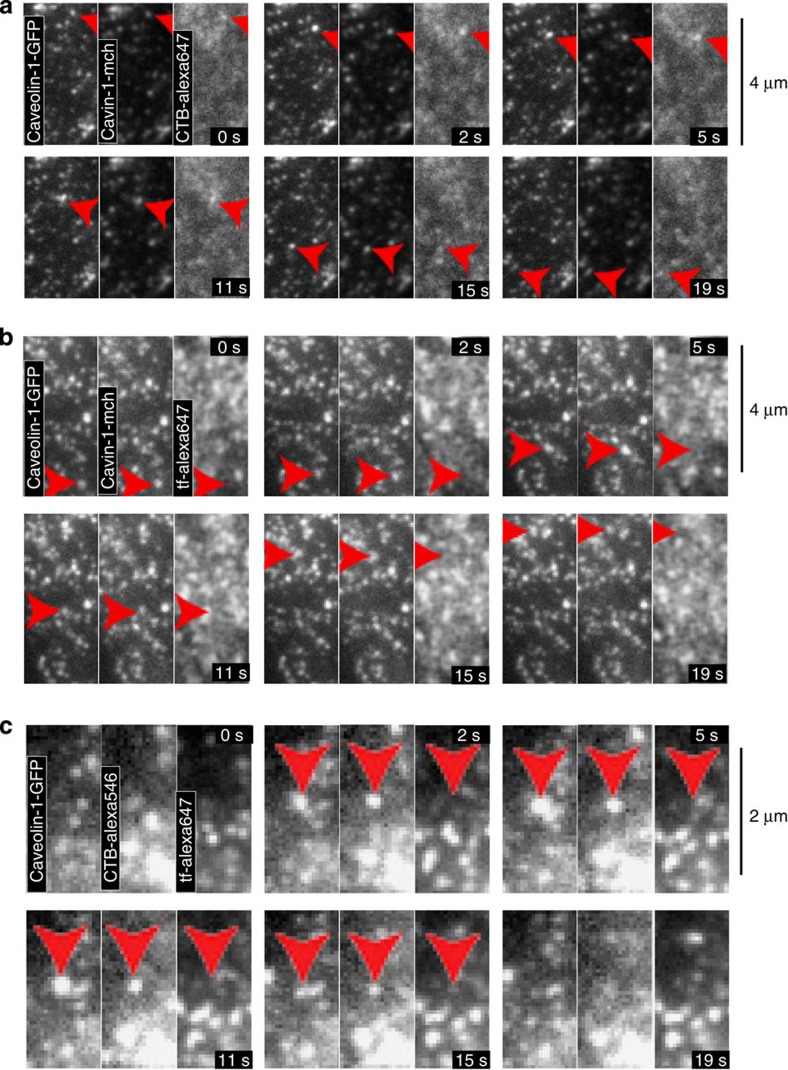
Dynamic endosomes containing genome-edited caveolin-1-GFP and cavin-1-mCh. (**a**) Time-lapse TIR imaging of live genome-edited cells expressing caveolin-1-GFP and cavin-1-mCherry incubated with alexa647 conjugated CTB (1 μg ml^−1^) for 15 min at 37 °C to allow internalization. Arrows indicate mobile CTB-positive structure. (**b**) Time-lapse TIR imaging of live genome-edited cells expressing caveolin-1-GFP and cavin-1-mCherry incubated with alexa647 conjugated transferrin (5 μg ml^−1^) for 15 min at 37 °C to allow internalization. Arrows indicate mobile structure. Mobile structures are not labelled with transferrin. (**c**) Time-lapse TIR imaging of live genome-edited cells expressing caveolin-1-GFP incubated with transferrin-alexa647 and CTB-alexa546 for 15 min at 37 °C to allow internalization. Arrows indicate mobile structure, which appears and disappears in the TIR field. Mobile structures are not labelled with transferrin.

Time-lapse imaging was used to confirm that motile caveolin-1-GFP and cavin-1-mCherry-positive vesicles contain endocytosed cargo, and hence by definition are endosomes. Cholera toxin B subunit (CTB), which binds to the glycosphingolipid GM1, is a potential marker for caveolar endocytosis^17^,^53^. Caveolin-1-GFP, cavin-1-mCherry cells were labelled with CTB-alexa647 for 15 min, and then imaged using TIR illumination. Motile structures containing all three markers that translocated long distances through the cell and disappeared and appeared within the TIR field were readily detected (Fig. 3a, Supplementary Movie 5). After 15-min uptake of CTB over 80% of clearly discernable motile structures marked by caveolin-1-GFP and cavin-1-mCherry also contained detectable CTB. For the reasons outlined above, these are likely to be discontinuous with the plasma membrane and hence represent endosomes of some kind. Time-lapse imaging of motile caveolin-1-GFP and cavin-1-mCherry-positive vesicles in cells loaded with fluorescent transferrin additionally suggested that these vesicles do not always contain transferrin. These caveolar endosomes may therefore be distinct from the early endosomes through which transferrin is transported (Fig. 3b, Supplementary Movie 6). This was confirmed by time-lapse imaging of caveolin-1-GFP cells loaded continuously for 20 min with CTB and transferrin. Structures containing caveolin-1-GFP and CTB but not transferrin could be detected appearing and disappearing within the TIR field, and hence unlikely to be continuous with the plasma membrane (Fig. 3c, Supplementary Movie 7).

**Figure 4:**
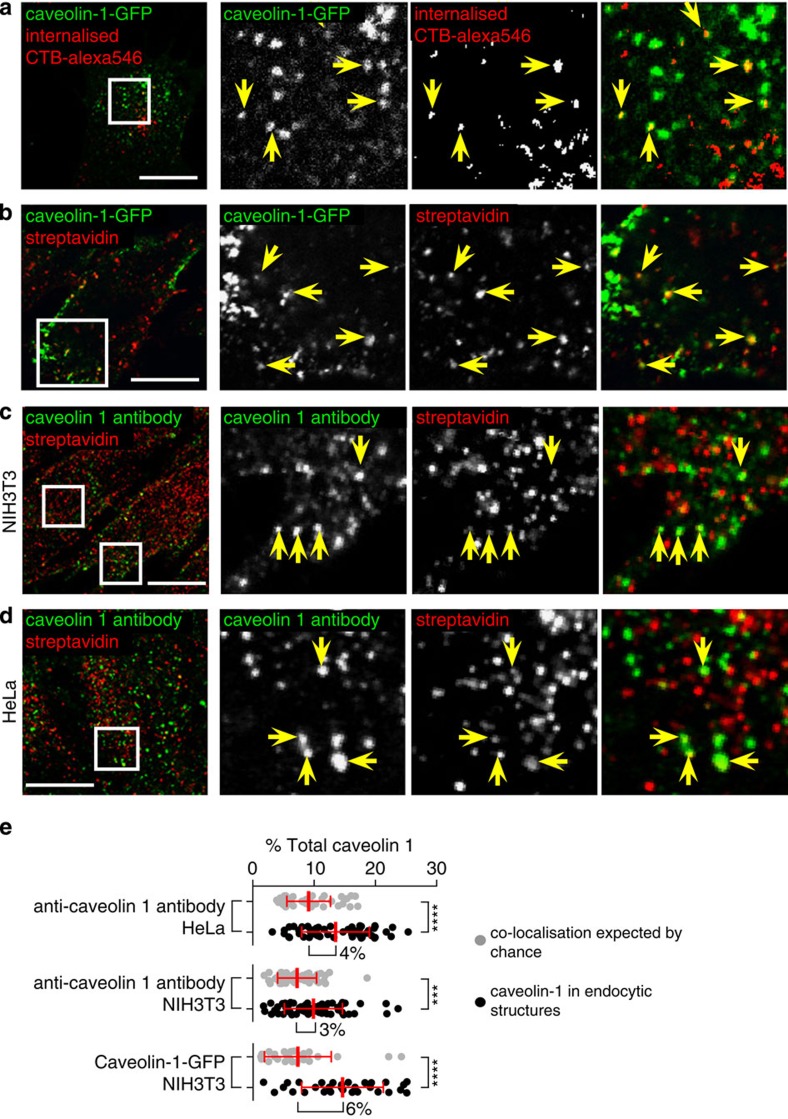
Genome-edited (5%) caveolin-1-GFP is present in endosomes. (**a**) Confocal image to show co-localization between caveolin-1-GFP and internalized CTB. The image showing internalized CTB is calculated by subtraction of images generated by consecutive labelling to separately detect total CTB after 15 min uptake and surface-accessible CTB only. Raw data and further explanation is given in Supplementary Figure 4. Arrows indicate co-localization in internalized structures. Bar is 10 μ, the white box delineates the region shown at higher magnification in the 3 right-hand panels. (**b**) Confocal image of internalized sulfo-NHS-SS-Biotin labelling surface free amine groups, after 15 min uptake and removal of extracellular biotin using the membrane impermeant reducing agent MESNA. Biotin was detected with fluorescent streptavidin. Controls in Supplementary Figure 5. Arrows indicate co-localization between caveolin-1-GFP and biotin in internalized structures. Bar is 10 μ, the white box delineates the region shown at higher magnification in the three right-hand panels. (**c**) Confocal image of non-genome-edited NIH3T3 cell showing internalized sulfo-NHS-SS-Biotin, after 15 min uptake and removal of extracellular biotin using the membrane impermeant reducing agent MESNA, and labelling with anti-caveolin-1 antibody. Arrows indicate co-localization between caveolin 1 and biotin in internalized structures. Bar is 10 μ, the white box delineates the region shown at higher magnification in the three right-hand panels. (**d**) As C, but image is of a HeLa cell. (**e**) Quantification of co-localization between the caveolin 1, caveolin-1-GFP and internalized protein labelled with sulfo-NHS-SS-biotin and MESNA treatment. Internalization was for 15 min. To establish empirically the degree of overlap between internalized protein and relevant marker expected by chance, quantification was carried out both with the images in the correct register, and also with one channel manually offset 500 nm from the other. Quantification of offset images is shown as grey dots, correct registration as black dots, statistically significant increase in co-localization with images in the correct register indicates biologically significant co-localization. Bars are mean and s.d., each point is one cell region.

Further assays were used to ask whether caveolin-1-GFP is present in endosomes that are not continuous with the plasma membrane. First, genome-edited NIH3T3 cells where caveolin-1-GFP (but not cavin-1-mCherry) is expressed from the *caveolin 1* locus as previously, were labelled with CTB-alexa546 and then CTB-alexa647 using a consecutive labelling approach, so that inaccessibility to the second label reflects internalization into the cell (Supplementary Fig. 4A, 4B). This allowed calculation of an image representing only internalized CTB (Fig. 4a and Supplementary Fig. 4C). Internalized CTB clearly co-localized with caveolin-1-GFP in a proportion of structures (Fig. 4a). The difference map representing only internalized CTB was used to produce a mask to reveal caveolin-1-GFP definitely inaccessible to the second label (Supplementary Fig. 4C). In cells analysed in this way the mean proportion of total caveolin-1-GFP found in endosomes was 4%, with a range from <1 to 9%.

In a completely separate approach, we used biotinylation of total surface-accessible free amine groups with sulfo-NHS-SS-biotin, and subsequent treatment with the membrane-impermeant reducing agent MESNA to remove external biotin, as an assay for endocytosis of free amine-containing molecules (which will include both proteins and glycolipids) from the plasma membrane (Fig. 4b, controls in Supplementary Fig. 5)^41^. After 15 min of uptake, co-localization between internalized biotin and caveolin-1-GFP in punctate endosomes was observed (Fig. 4b). It was important to confirm that the presence of caveolin-1-GFP on endosomes is not merely a consequence of GFP tagging. The surface biotinylation plus MESNA approach was applied to non-genome-edited NIH3T3 cells, and to HeLa cells. After 15 min of internalization cells were fixed and labelled with anti-caveolin-1 antibodies. Again, co-localization between internalized biotin and caveolin 1 in intracellular structures could be observed (Fig. 4c,d).

The proportion of caveolin 1 or caveolin-1-GFP-positive structures that are endosomes by the criterion of uptake of biotinylated plasma membrane components is clearly small, and both biotin-positive endosomes and caveolin-1-positive puncta are abundant in cells. This is consistent with our recent experiments arguing that the large majority of all endocytic vesicles are produced via budding of clathrin-coated pits^41^. It was therefore important to develop methods to quantify co-localization that account for possible random overlap due to the high abundance of both classes of structure. We used a pixel-mask based approach to calculate the proportion of caveolin-1-positive pixels that also contain internalized biotin in both unadjusted images and in images where the caveolin 1 channel was artificially offset by 500 nm. This provided an empirical way to calculate the amount of co-localization occurring by chance. This approach confirmed that there is indeed specific co-localization between internalized biotin and both caveolin 1 antibody staining and caveolin-1-GFP in endosomes, and suggested that around 3–5% of all caveolin 1 is present in these endosomes (Fig. 4e).

### Characterization of caveolar endosomes

**Figure 5:**
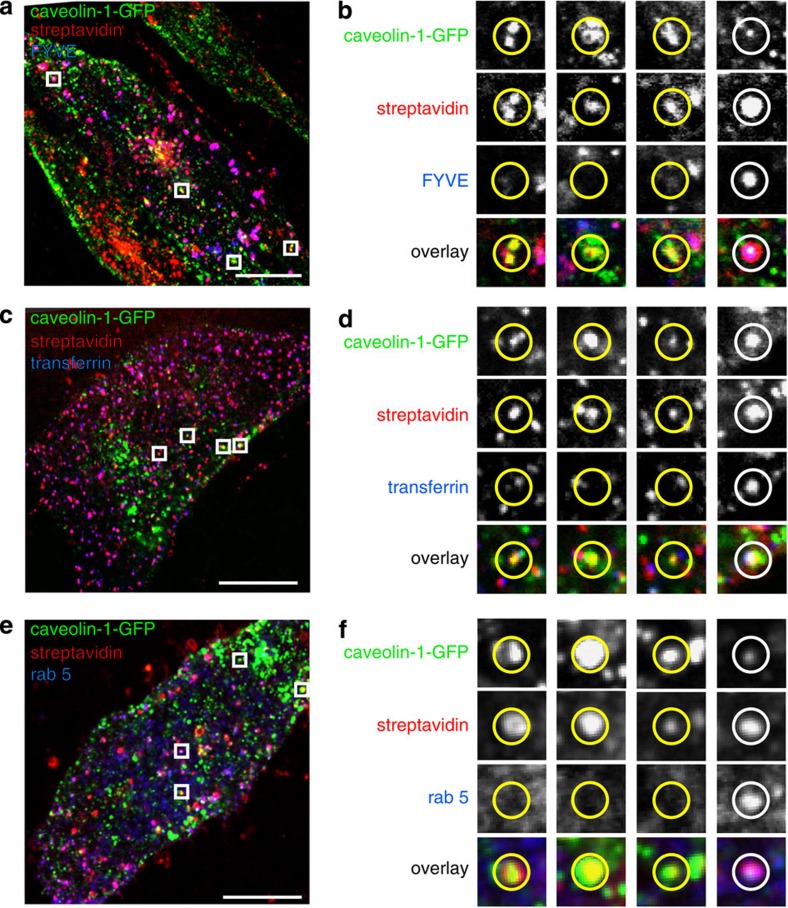
Caveolin-positive endosomes include both classical early endosomes and separate organelles. (**a**) Confocal image of an NIH3T3 cell expressing genome-edited caveolin-1-GFP and FYVE-mCherry, labelled with streptavidin to detect internalized sulfo-NHS-SS-biotin after 15 min uptake. The boxes denote regions shown in **b**. Bar is 15 μm. (**b**) Examples of caveolin-1-GFP-positive endosomes that do not recruit FYVE-mCherry, taken from the cell shown in A, yellow circles. One example of an endosome that does recruit FYVE-mCherry is also shown, white circle. (**c**) Confocal image of an NIH3T3 cell expressing genome-edited caveolin-1-GFP, labelled with streptavidin to detect internalized sulfo-NHS-SS-biotin after 15 min uptake, and with transferrin internalized continuously for the same length of time. The boxes denote regions shown in **d**. Bar is 15 μm. (**d**) Examples of caveolin-1-GFP-positive endosomes that do not contain transferrin, yellow circles, and one endosome that does contain transferrin, white circle, taken from the cell shown in **c**. (**e**) Confocal image of an NIH3T3 cell expressing genome-edited caveolin-1-GFP and Rab5-mCherry, labelled with streptavidin to detect internalized sulfo-NHS-SS-biotin after 15 min uptake. The boxes denote regions shown in **b**. Bar is 15 μm. (**f**) Examples of caveolin-1-GFP-positive endosomes that do not recruit Rab5-mCherry, taken from the cell shown in A, yellow circles. One example of an endosome that does recruit Rab5-mCherry is also shown, white circle.

We sought to ascertain the identity of the endosomal membranes containing endogenous caveolin-1-GFP and caveolin 1. Overexpressed caveolin-1-GFP is found in classical early endosomes^12^,^31^,^36^. Defining characteristics of classical early endosomes include the presence of internalized transferrin, recruitment of the small GTPase Rab5, and importantly production of the lipid PI3P^54^. Using internalization of sulfo-NHS-SS-biotin and MESNA treatment to identify endogenous caveolin-1-GFP-positive endosomes as previously, we found that many of these endosomes clearly did not recruit the PI3P-binding FYVE domain (Fig. 5a,b), did not contain transferrin after 15-min continuous labelling (Fig. 5c,d), and did not recruit Rab5-mCherry (Fig. 5e,f). It should be noted that in all cases there were also triple-labelled endosomes present, so caveolae are present in both caveolar endosomes and in classical early endosomes defined by PI3P, transferrin and Rab5.

### Caveolar budding from the plasma membrane

We designed experiments to address the question of whether budding of caveolae from the plasma membrane mediates delivery of plasma membrane components to caveolae-containing endosomal compartments. The alternative possibility was that caveolae reside in or traffic via endosomes, but are taken up from the cell surface using another mechanism than direct budding of the caveolar structure itself.

**Figure 6:**
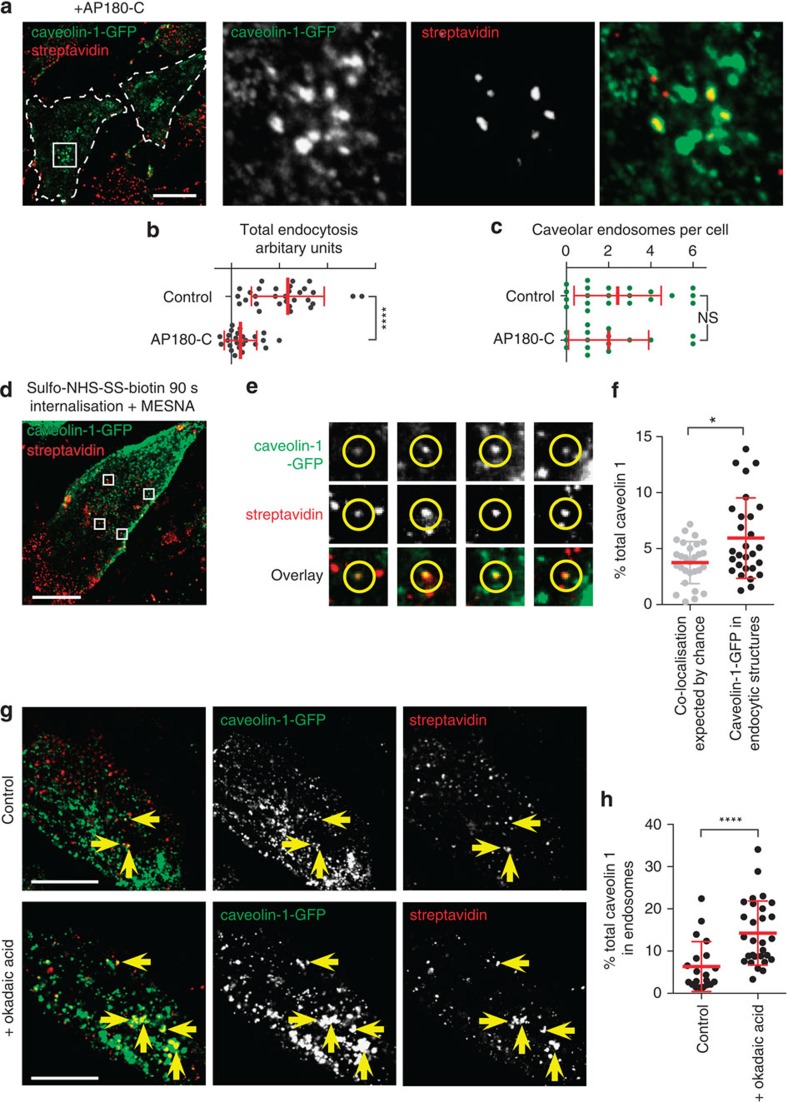
Budding of caveolae from the plasma membrane delivers endocytosed cargo to caveolar endosomes. (**a**) Genome-edited caveolin-1-GFP cells were transfected with myc-AP180-C. Cells were labelled with streptavidin to detect internalized sulfo-NHS-SS-biotin after 15 min uptake and MESNA treatment. Transfected cells are outlined with a dashed line. The region shown by a white box is shown in the magnified panels to the right-hand side. (**b**) Quantification of total endocytosis of sulfo-NHS-SS-biotin, detected by streptavidin labelling after MESNA treatment, in control and AP180-C expressing cells. Bars are mean and s.d. (**c**) Quantification of caveolar endosomes abundance. Caveolar endosomes were scored by eye as structures clearly containing both caveolin-1-GFP and internalized sulfo-NHS-SS-biotin as shown in A. Bars are mean and s.d. (**d**) Confocal image of an NIH3T3 cell expressing genome-edited caveolin-1-GFP, labelled with streptavidin to detect internalized sulfo-NHS-SS-biotin after 90 s uptake. The boxes denote regions shown in E. Bar is 15 μm. (**e**) Individual caveolar endocytic vesicles after 90 s uptake, taken from **d**. (**f**) Quantification of co-localization between caveolin-1-GFP and internalized protein labelled with sulfo-NHS-SS-biotin and MESNA treatment. Internalization was for 90 s. To establish empirically the degree of overlap between internalized protein and relevant marker expected by chance, quantification was carried out both with the images in the correct register and also with one channel manually offset 500 nm from the other. Quantification of offset images is shown as grey dots, correct registration as black dots, statistically significant increase in co-localization with images in the correct register indicates biologically significant co-localization. Bars are mean and s.d., each point is one cell region. (**g**) Confocal image of an NIH3T3 cells expressing genome-edited caveolin-1-GFP, labelled with streptavidin to detect internalized sulfo-NHS-SS-biotin after 15 min uptake. Cells were treated with 500 nM okadaic acid as shown. Arrows indicate co-localization in caveolar endosomes. (**h**) Quantification of co-localization between caveolin-1-GFP and internalized protein labelled with sulfo-NHS-SS-biotin and MESNa treatment. Internalization was for 15 min, cells were treated with 500 nM okadaic acid.

First, we asked whether delivery of endocytosed cargo to caveolar endosomes is clathrin-dependent. Inhibition of clathrin-coated pits by overexpressing AP180-C significantly reduced total endocytosis, but did not block delivery of biotinylated substrates to caveolar endosomes (Fig. 6a–c). This suggests that uptake to caveolar endosomes is indeed likely to be clathrin independent.

Second, we labelled cells with sulfo-NHS-SS-biotin and then allowed internalization to procede for just 90 s. This will lead to labelling of primary endocytic vesicles that have just budded from the plasma membrane. Even after just 90 s, significant co-localization between internalized biotin and caveolin-1-GFP in individual punctate structures was detected (Fig. 6d–f). We conclude that caveolar endocytosis is mediated by clathrin-independent budding of caveolae.

If endocytosis to caveolin-positive endosomes is mediated by budding of caveolae from the plasma membrane, then perturbations known to upregulate caveolar budding would be predicted to increase the abundance of these endosomes. Okadaic acid increases caveolar budding^40^,^55^, and treatment of genome-edited caveolin-1-GFP cells with 500-nm okadaic acid caused a clear increase in the proportion of caveolin-1-GFP that contains internalized sulfo-NHS-SS-biotin (Fig. 6g,h). Therefore, budding of caveolae from the plasma membrane is likely to be the means by which endocytosed membrane is delivered to caveolar endosomes.

### Caveolae exclude proteins with transmembrane domains

**Figure 7:**
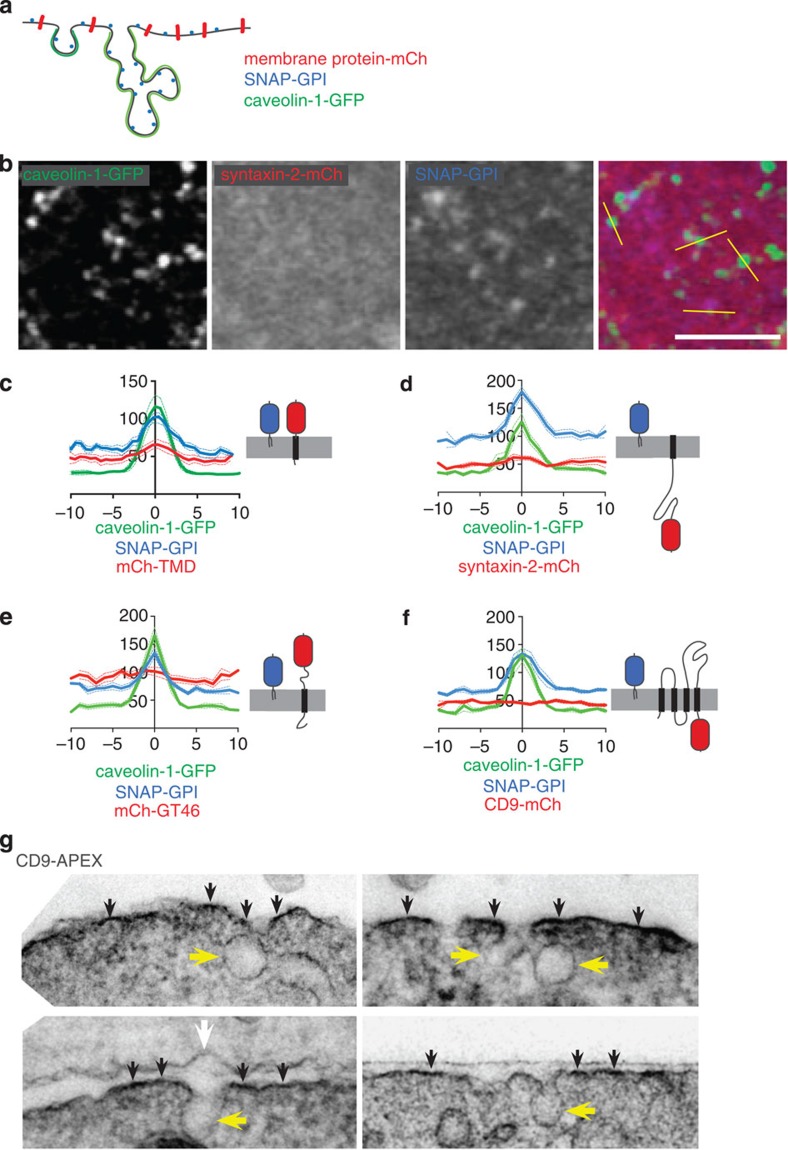
Caveolae exclude bulk membrane proteins. (**a**) Cartoon to illustrate how increased membrane convolution within caveolae will cause local increase in fluorescence intensity in images of proteins with a uniform distribution per unit area of membrane, such as GPI-linked proteins. In contrast, proteins that are excluded from caveolae will appear at uniform fluorescence intensity across the region containing caveolae. (**b**) Confocal image of NIH3T3 cells expressing caveolin-1-GFP, transiently transfected for 24 h with syntaxin-2-mCherry and SNAP–GPI. Cells were incubated at 4 °C for 15 min with membrane impermeant SNAP Cell Surface Reagent 647 to label cell surface-associated GPI proteins before fixation and confocal imaging. Bar is 2 μ. (**c**,**d**,**e**,**f**) Quantification of intensity profiles across caveolin-1-GFP-positive regions as shown in **b**. The intensity of caveolin-1-GFP, SNAP-GPI labelled as in A, and the fluorescent membrane protein shown in red, was quantified for 20 different puncta, and plotted as mean+/− s.e.m. Distance (*x* axis) and fluorescence intensities (y axis) are in arbitrary units. (**g**) Electron micrographs of cells expressing CD9-APEX. Reactive oxygen generated by the APEX tag triggers polymerization of DAB, leading to the electron-dense deposit all along the plasma membrane. The white arrow indicates plasma membrane of a non-transfected cell. Yellow arrows indicate caveolar membrane bulb, and black arrows highlight the extra electron density specifically generated by the APEX tag.

Prompted by our analysis of a specific protein coat all around the caveolar bulb^4^, we hypothesized that this coat could modify the local protein complement of the plasma membrane, for example, via steric exclusion of proteins with cytosolic domains. Multiple studies using both electron microscopy and FRET measurements indicate that GPI-linked proteins have the same concentration per unit membrane in caveolae as in the rest of the plasma membrane, implying that this class of proteins can be used as a reporter of the total amount of membrane present in a given area^56^,^57^,^58^,^59^ (Fig. 7a). The intensity of signal from fluorescently labelled GPI-linked protein (SNAP–GPI) at the cell surface was higher in caveolae than in the rest of the membrane, reflecting the complex and convoluted membrane morphology of clusters of caveolae (Fig. 7a,b). To compare the GPI-linked protein with an analogous minimal protein anchored to the plasma membrane with a transmembrane helix we used a construct composed of a signal peptide, mCherry, an artificial transmembrane domain composed of seven repeats of LVA, and a cytosolic FLAG epitope (mCherry-TMD). Ratiometric imaging revealed that this protein is clearly less abundant within caveolae than SNAP–GPI, implying that even a single transmembrane helix and cytosolic domain of just ten amino-acid residues is depleted within the caveolar bulb (Fig. 7c). Consistent with this, ratiometric imaging confirmed that three further proteins, selected to have type I, type II and multiple transmembrane domain topologies, were also all depleted within caveolae relative to SNAP–GPI (Fig. 7d–f). These observations are best explained by exclusion of proteins with transmembrane domains from caveolae.

We used electron microscopy to test the conclusion that transmembrane proteins are excluded from caveolae more directly. CD9 was fused to APEX, a modified form of ascorbate peroxidase that can be used as a genetically encoded tag for electron microscopy^60^. Polymerization of diaminobenzidine by reactive oxygen species produced by APEX led to an osmiophilic deposit along the plasma membrane in transfected cells, and this was clearly more electron dense than background staining of membranes by osmium itself (Fig. 7g). Electron density produced by APEX and diaminobenzidine was excluded from caveolae (Fig. 7g). This confirms that CD9 is excluded from caveolae and thereby confirms our interpretation of the light microscopy data presented above.

### Caveolae control sorting of excess glycosphingolipid

The observation that caveolae are relatively depleted of transmembrane proteins, together with previous reports of glycosphingolipid transport within caveolae, is consistent with a role in lipid trafficking^1^,^26^. To determine the cellular function of caveolae, *caveolin 1−/−* MEFs were loaded with an exogenous fluorescent analogues (BODIPY conjugates) of GM1 and lactosyl ceramide by addition of the lipids bound to albumin^43^,^45^,^61^. The amount of both lipids incorporated into cells on ice was not appreciably different between control and *caveolin 1−/−* cells (Supplementary Fig. 7A). Moreover, the subcellular distribution of both lipids after short periods of uptake (for example 15 min, Supplementary Fig. 7B) was not notably different in the absence of *caveolin 1*. These data, consistent with several previous studies^62^,^63^, the low fraction of CTB internalized into caveolin-1-positive endosomes, and the low mobility of caveolae in FRAP experiments, all suggest that, in fact, a relatively low fraction of the total endocytosis of glycosphingolipid takes place in caveolae.

**Figure 8:**
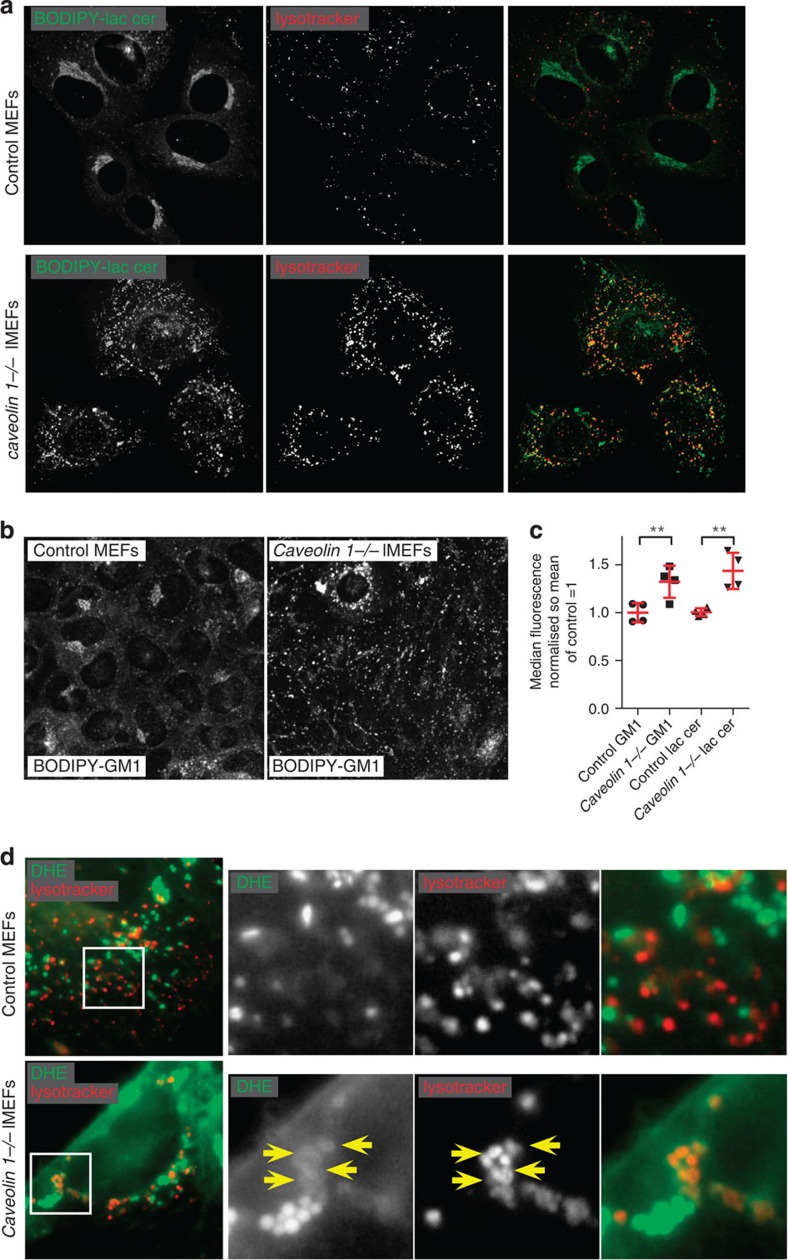
Exogenous glycosphingolipid accumulates in lysosomes of *caveolin 1−/−* cells. (**a**) Confocal images of live immortalized MEFs from wild-type or *caveolin-1 −/−* animals, loaded for 3 h at 37 °C with 5 μM BODIPY-Lactosyl ceramide complexed with BSA. Prior to imaging, cells were labelled with 20 nM of LysoTracker Deep Red according to manufacturer instructions. The images are representative of two different clones of control or *caveolin 1−/−* cells, in at least three independent experiments. (**b**) Confocal images of live immortalized MEFs from wild-type or *caveolin 1−/−* animals, loaded for 3 h at 37 °C with 5 μM BODIPY-Ganglioside M1 complexed with BSA, then washed, incubated for another 60 min at 37 °C. (**c**) Quantification of GM1 or LacCer amounts using flow cytometry analysis. Live immortalized MEFs from wild type or *caveolin-1−/−* animals were loaded for 3 h at 37 °C with 5 μM BODIPY-Lactosyl ceramide or BODIPY-GM1 and then washed, incubated for 60 min at 37 °C, and the amount of lipids left in the cells was quantified by flow cytometry. Two different clones of control or *caveolin 1−/−* cells were tested in every experiment, and graph shows mean+/− s.e.m. of two independent experiments. (**d**) Live immortalized MEFs from wild type or *caveolin-1−/−* animals were loaded for 3 h in serum-free medium at 37 °C with dehydroergosterol:methyl-beta-cyclodextrin complexes at 30 mM methyl-beta-cyclodextrin. Cells were washed with 10% serum-supplemented medium, incubated for 60 min at 37 °C, and then imaged.

Given the above, we reasoned that the effects of loss of caveolae on glycosphingolipid homeostasis may have much slower kinetics than total endocytosis, and may only be apparent when cells are loaded with excess lipid. Cells were loaded with BODIPY-GM1 or BODIPY-lactosyl ceramide for 3 h, and then chased for 1 h at 37 °C. The intracellular distribution of both lipid analogues was then strikingly different in the *caveolin 1−/−* cells. In control cells they accumulated in a perinuclear structure that previous studies imply is likely to represent the Golgi apparatus and potentially recycling endosomes (Fig. 8a,b)^45^, but in knockout cells this perinuclear pool was hard to detect, and instead BODIPY was observed in bright puncta that labelling with the pH-sensitive dye lysotracker revealed to be late endosomes or lysosomes (Fig. 8a). This was accompanied by an increase in the total amount of lipid present in the cells, as assayed by flow cytometry (Fig. 8c). There was no detectable alteration in the metabolism of BODIPY-GM1 or of BODIPY-lactosyl ceramide in *caveolin 1−/−* cells (Supplementary Fig. 8). Therefore, under conditions of extra load of glycosphingolipid in the plasma membrane, loss of *caveolin 1* function causes missorting of internalized glycosphingolipid to lysosomes and accumulation of lipid in this location.

Glycosphingolipids are thought to be trafficked in association with membrane cholesterol^43^,^64^, so we asked whether sterols are also likely to be mis-sorted in *caveolin 1−/−* cells. The cholesterol analogue dehydroergosterol (DHE) is fluorescent, and so provides a way to monitor cellular response to increased sterol loading^65^. Control and *caveolin 1−/−* cells were loaded with DHE from DHE-cyclodextrin complexes^65^ for 3 h, and then chased for 1 h at 37 °C. In contrast to glycosphingolipids, DHE mainly accumulated in lipid droplets in cells of both genotypes (Fig. 8d). However, in the *caveolin 1−/−* cells DHE fluorescence was also clearly observed co-localizing with lysotracker (Fig. 8d). Therefore loss of caveolin 1 causes a fraction of internalized DHE to accumulate along with glycosphingolipids in lysosomes.

**Figure 9:**
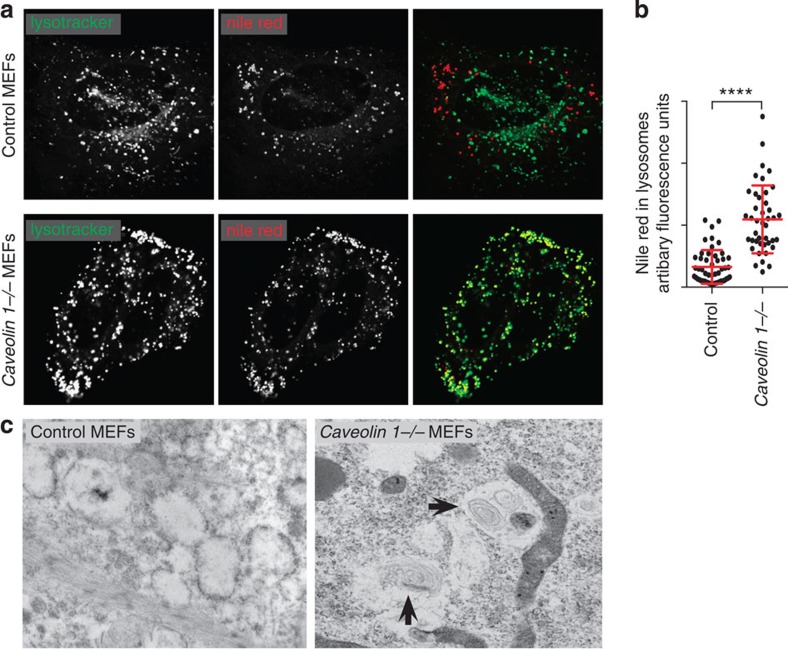
Lipid inclusions in lysosomes of *caveolin 1−/−* cells. (**a**) Immortalized MEFs from wild type or *caveolin 1−/−* animals were loaded for 3 h at 37 °C with 5 μM of non-fluorescent GM1 in the presence of 0.2% BSA. Prior to imaging cells were labelled with nile red (0.5 μM) and LysoTracker Deep Red (20 nM ) according to manufacturer instructions. (**b**) Quantification of nile red in lysosomes. Each dot represents one cell, lysotracker-positive pixels were isolated by thresholding as described in Methods. Bars are mean and s.e.m. (**c**) Electron micrographs of GM1 loaded control and *caveolin 1−/−* MEFs showing lipid inclusions in lysosomes from *caveolin 1−/−* cells (highlighted with black arrows).

Accumulation of glycosphingolipid in lysosomes could reflect buildup of lysosomal phospholipid inclusions, as happens in the absence of the lysosomal acid sphingomyelinase in Niemann–Pick disease. This is indeed the case, because when both control *and caveolin 1−/−* cells were loaded with GM1 (without BODIPY) there was a clear and marked accumulation of nile red-positive inclusions within lysosomes in the *caveolin 1−/−* cells (Fig. 9a,b). As general hydrolytic activity of lysosomes was not detectably impaired (Supplementary Fig. 9), this is likely to reflect saturation of the capacity of lysosomes to degrade extra glycosphingolipid consequent to missorting to lysosomes in the absence of caveolin 1. In agreement with this, electron microscopy of *caveolin 1−/−* and control cells after loading with GM1 as above, revealed abundant multilamellar inclusions in lysosomes of the *caveolin 1−/−* cells (Fig. 9c).

## Discussion

We have used genome-edited NIH3T3 cells to characterize the endosomal dynamics of caveolae. Both caveolin-1-GFP and cavin-1-mCherry, expressed from the relevant endogenous loci, are present in specific intracellular endosomes. The fact that caveolin 1 and cavin 1 are both present in these structures implies that they are clusters of caveolae, as assembly of these two proteins into a caveolar coat complex is associated with formation of the distinctive membrane morphology of caveolae^4^,^6^. Caveolar endosomes have been described previously as caveosomes^32^,^37^, but we have avoided this term. Endosomes defined by the presence of caveolae are likely to be heterogeneous, including both transferrin-positive early endosomes and separate endosomal structures. It is possible that interlinked clusters of caveolae may bud as a unit from the plasma membrane, and this would generate primary endocytic vesicles much larger than single caveolae.

To characterize the properites of caveolar endosomes and endocytosis, we have applied a range of perturbations to our genome-edited cells. In agreement with previous studies, we find that caveolar endocytosis is clathrin independent, in that it is not blocked by overexpression of AP180-C and it leads to production of endocytic vesicles at very short periods of uptake that are devoid of transferrin^62^. Similarly, we find that overexpression of an ATPase-active mutant of EHD2 immobilizes caveolae^12^,^13^.

Overexpression of caveolin-1-GFP is likely to lead to multiple artifacts, including the presence of caveolin-1-GFP in non-caveolar plasma membrane structures and accumulation of the overexpressed protein in late endosomes^36^,^37^. Genome editing provides a means to avoid overexpression, but there is still a risk that addition of the GFP moiety will perturb caveolin 1 function. Three main observations argue that this is not the case. First, endogenous caveolin 1 is found on endosomes with the same frequency as caveolin-1-GFP expressed by genome editing. Second, genome-edited caveolin-1-GFP and cavin-1-mCherry co-localize remarkably completely, showing that the tagged proteins can assemble into the correct protein complex^4^. Third, although we have chosen not to show multiple duplicated experiments here, cavin-1-mCherry, expressed by genome editing in a cell line in which caveolin 1 is not tagged with GFP, is also present on dynamic endosomes that can exclude transferrin. Observation of caveolar endosomes is then not contingent on the presence of caveolin-1-GFP.

The composition of the plasma membrane within the caveolar bulb will be critical for the function of caveolae in intracellular membrane trafficking. We show that caveolae are likely to exclude bulk plasma membrane proteins with transmembrane and cytosolic domains. One may imagine that this exclusion could be generated by the very high radius of curvature at the neck of caveolar membranes, or by the presence of the caveolar coat complex closely associated with the surface of the caveolar bulb^4^. The presence of GPI-anchored proteins in caveolae shows that lipid anchors and hence lipids can diffuse into the caveolar bulb in the outer leaflet of the plasma membrane^57^. These observations provide a plausible explanation as to why general protein cargoes for caveolar endocytosis have been lacking.

Phenotypes of animals lacking caveolae include profound lipodystrophy and changes in adipocyte function^26^, and hence argue strongly for a role in lipid homeostasis. Caveolae determine plasma membrane lipid composition^44^, and have been associated with trafficking of glycosphingolipids^43^,^61^. A role in vesicle transport of membrane lipid of is compatible with recent data proposing a role for caveolae in membrane repair, internalizing damaged regions of the plasma membrane^22^. Such a function is, after all, a specialized form of lipid transport. Our new data show that caveolae are well adapted for a function in vesicular transport specifically of membrane lipids through active exclusion of bulk membrane proteins. The rate of budding of caveolae from the plasma membrane is likely to be low relative to that of coated pits^66^, and it is not clear that any specific lipid species is ever highly enriched within caveolae. The data are then consistent with a model in which relatively slow caveolar endocytosis allows delivery of internalized excess glycosphingolipid to the ER–Golgi system, where there are enzymes for metabolism of these lipids. In the absence of caveolae, access to the Golgi is reduced and the glycosphingolipids instead accumulate in lysosomes, saturating the ability of lysosomes to break down these types of lipid. Further experiments will be required to assess the *in vivo* significance of the altered pattern of lipid transport that we observe in *caveolin 1−/−* cultured cells.

## Methods

### Antibodies and reagents

The following antibodies were used: mouse anti-GFP (Roche 11814460001, dilution 1:5,000), rabbit anti-cavin1 (Abcam ab48824, dilution 1:2,000), rabbit anti-caveolin 1 (BD Biosciences 610060, dilution 1:5,000), goat polyclonal anti-EHD2 (Abcam ab23935, dilution 1:1,000), rabbit anti-RFP (MBL PM005, dilution 1:2,000), rabbit anti-Cholera toxin beta subunit (Abcam ab34992, dilution 1:300) and rabbit anti-actin (Sigma C3956, dilution 1:5,000). Horse radish peroxidase (HRP)-conjugated secondary antibodies were from DAKO and fluorophore-conjugated antibodies and streptavidin were from Molecular Probes (Invitrogen).

The reagents used in this study are: cholera toxin B subunit conjugated to HRP (Molecular Probes, C3478) or alexa fluor 555 (Molecular Probes, C34776) or alexa fluor 647 (Molecular Probes, C34778); alexa fluor 647-conjugated human transferrin (Invitrogen, T23366); SNAP Cell surface Reagent 549 (New England Biolabs, S9112S); cyclohexamide (Santa-Cruz, sc-3508); okadaic acid (Santa-Cruz, sc-95060);and Magic Red Cathepsin L kit (MR-FR2, Immunochemistry Technologies).

### Cell culture

HeLa (ATCC) and NIH3T3 cells (a gift from the lab of Julian Sale, MRC-LMB) were grown in DMEM (Gibco) supplemented with penicillin and streptomycin, and 10% fetal calf serum. For overexpression experiments HeLa or NIH3T3 cells were transfected using FugeneHD (Promega), in accordance with the manufacturer's instructions.

### DNA constructs

Caveolin-1-GFP and Cavin-1-mCherry plasmid constructs for transient transfection have been described previously^7^,^14^. Rab5-mCherry, and Rab7-mCherry and Syntaxin2-mCherry were gifts from A. Ludwig, Nanyang Technological University, Singapore. YFP-GPI, GFP-CD9 and LYFPGT46 were described previously^67^. LmCherryGT46 was produced by exchanging YFP with mCherry (sourced from pmCherry-N1, Clontech). CD9-mCherry was produced by inserting CD9 into XhoI and BamHI site of pmCherry-N1 (Clontech). APEX-GFP-CD9 was produced by inserting APEX cDNA at N-terminus of GFP-CD9 using Gibson assembly. Mouse CD36-mCherry was produced by inserting mouse CD36 cDNA (Image Clone LLAM 8510 D02, Source Biosience) into XhoI and HindIII sites of mCherry-n1 (Clontech). Dynamin splice variants and mutants were a kind gift from M. Frick, Ulm University. mCherry-TMD was a kind gift from J. Claessen, MRC-LMB. EHD2 plasmids were as described^14^.

### Genome editing

TALE nucleases (TAL Effector Nucleases) designed to cleave precisely at or in the immediate vicinity of the stop codon of the mouse *caveolin 1* gene were produced by LabOmics. Cleavage activity was validated using a cell-based surrogate reporter system^68^ that was also provided by LabOmics. Cas9 used in this study was applied as described as^49^, and obtained from Addgene. To find potential guide RNA sequence candidates we used the software E-CRISP, and then these sequences were further screened manually via BLAST to determine the best candidate. The chosen 23 nucleotide sequence (5′- GCTGGAGATCACCGAGGAGTCGG -3′) was used to generate a plasmid containing a 455 bp fragment (synthesized as gBlock from IDT) bearing all components necessary for guide RNA expression, again as described^49^.

Donor DNA constructs containing flanking regions for gap repair by homolous recombination and the appropriate fluorescent protein DNA were produced as follows. Approximately 1.1 kb of genomic DNA sequence on either side of the *caveolin 1* and *cavin 1* stop codons were amplified from appropriate BACs using primers listed in Table 1. DNAs coding for GFP or mCherry, originated from pEGFP-N1 or pCherry-N1 (Clontech) were amplified using the primers listed at Table 1. For targeting of *caveolin 1*, the *GFP* gene and left and right homologous arms were fused using Fusion PCR technique^69^, with the stop codon deleted from *caveolin 1* and a linker between caveolin 1 and GFP identical to that described previously^32^. XhoI/ NotI restriction sites were introduced into the fused product, which was inserted into Xhoi/ NotI sites of pBluescript SK−/−. Similarly, for targeting of *cavin 1*, in the donor construct in the stop codon of the *cavin 1* gene was deleted and cDNA for mCherry was fused the same linker as described^7^, using Gibson Mater kit assembly (New England BioLabs) according to the manufacturer's instructions.

For generation of genome-edited NIH3T3 cell lines, TALE nucleases or cas9/guiding RNA plasmids and donor plasmids were transfected into cells using Neon transfection system (Invitrogen). After transfection, cells were cultured for 5 days to recover and express the protein of interest. Depending on the experiment, recovered cells were sorted for GFP-positive or RFP- and GFP-positive signals using a Sony iCyt Synergy Dual Channel High Speed Cell sorter or Beckman Coulter MoFlo High Speed Cell sorter to obtain populations of positive cells. Correct gene targeting was determined by PCR, and by Western blotting as shown in Fig. 1a.

### Electron microscopy

Genome-edited NIH3T3 cells grown on MatTek dishes were fixed in 2.5% glutaraldehyde, 2% paraformaldehyde in 0.1 M cacodylate buffer pH 7.4 overnight at 4 °C. Cells were post-fixed in 1% osmium tetroxide for 1 h at 4 °C, washed and dehydrated through an increasing ethanol series and embedded in CY212 resin in dishes.

For ruthenium red staining of the cell surface, ruthenium red was added to 2.5% glutaraldehyde in 0.1 M cacodylate buffer at a concentration of 1 mg ml^−1^ for 1 h at room temperature, washed and fixed in 1% osmium tetroxide containing Ruthenium Red (1 mg ml^−1^) for 3 h in dark at room temperature. Ultrathin sections were cut both parallel to the cell substratum as well as perpendicular to it. Sections were viewed either unstained or stained with uranyl acetate and Reynolds lead citrate using an FEI Technai Spirit EM operated at 80 kV.

For pre-embedding immunolabelling, transfected HeLa cells were fixed in 4% paraformaldehyde in 0.1 M phosphate buffer overnight at 4 °C. After washing, cells were incubated in 0.1% sodium borohydride for 15 min, permeabilised in 0.03% saponin (10 min), washed and blocking serum (Aurion 905.002) added for 40 min before incubation in rabbit anti-GFP (Abcam 6556 ) 1:500 overnight at 4 °C. Cells were then incubated with F(ab)2 goat anti-rabbit ultrasmall gold conjugate (Aurion 100.166) for 2 h at room temperature, washed and fixed in 2.5% glutaraldehyde in 0.1 M phosphate buffer. Ultrasmall gold was then silver enhanced using R Gent SE-EM kit (Aurion 500.033), followed by 0.5% osmium tetroxide on ice and in the dark for 15 min. Cells were then processed from this point for conventional transmission electron microscopy as above. Ultrathin sections were stained with uranyl acetate and Reynolds lead citrate.

For Diaminobenzidine polymerization by HRP conjugated to CTB, NIH3T3 cells were incubated with HRP-conjugated CTB at 37 °C for different time points, and then placed on ice to stop internalization and washed in ice cold PBS. Cells were then incubated with Diaminobenzidine (Sigma) at a working concentration of 1 mg ml^−1^ ± 50 mM Ascorbic Acid in PBS for 20 min, washed briefly and fixed in 2.5% Glutaraldehyde in 0.1 M phosphate buffer overnight at 4 °C. After washing, cells were post-fixed in 1% Osmium Tetroxide in 0.1 M Phosphate buffer and processed for EM.

For detection of APEX-CD9 on cell surface, cells were transiently transfected with APEX-GFP-CD9 and processed according to the protocol described^60^.

### Cell imaging and immunofluorescence staining

Cells were grown on glass coverslips or in LabTek chambers overnight before fixation in 4% paraformaldehyde at room temperature for 7 min. After three washes with PBS, the cells were permeabilised with 0.1% Triton X-100/PBS. After brief washing with PBS, the cells were incubated with appropriate primary antibody for 60 min at room temperature. The cells were then washed with PBS for 15 min before incubation with secondary antibody for 30 min at room temperature. After three washes with PBS, the cells in LabTek chambers were visualized by confocal microscope, whereas coverslips were mounted on glass slides using ProLong Gold Antifade Reagent (Invitrogen). TIR images were acquired using an Olympus TIR microscope equipped with 488, 546 and 647 nm lasers and fitted with a 100 × , 1.45NA objective. Confocal images were acquired with a 63 × , 1.4 NA objective on a Zeiss 510 microscope.

Cells were loaded with dehydroergosterol using cyclodextrin: dehydroergosterol complexes as described^70^. Imaging of dedydroergosterol was carried out using a Nikon epifluorescence microscope equipped with a 100 × S-Fluor objective. Filtersets specifying excitation and emission wavelengths were again as described^65^,^70^.

### Photobleach experiments

FRAP studies were conducted on live NIH3T3 cells expressing either endogenous caveolin-1-GFP or overexpressed caveolin-1-GFP. Cells were seeded in LabTek chambers 24 h before experiment. Measurements were taken in growth media supplemented with 10 mM HEPES (Sigma), and the 37 °C temperature was controlled by heated stage incubator insert. FRAP experiments were performed on an inverted Zeiss LSM510 confocal microscope, using a 63 × , 1.4 NA objective. Three frames were taken before photobleaching to determine the average prebleach fluorescence at starting point. A defined region of interest (ROI; 8 μm diameter) was photobleached at full laser power. Recovery of fluorescence was monitored by scanning the ROI at low laser power in movies taken at rate of one frame per 2 s (120–180 frames per movie). The average fluorescence intensity in the ROI and the average background were determined from the images using LSM510 software. After subtracting the background, the fluorescence values were normalized to unbleached region with same ROI to correct for the loss in fluorescence caused by imaging. To be able to compare FRAP curves from different cells, the average fluorescence from three frames taken before photobleaching was set to 100% and the relative recovery in every cells was normalized to its initial level. Six to eight cells were imaged for every treatment in each experiment, and the final data represent mean ±s.e.m. from at least three independent experiments.

### Cell surface biotinylation and internalization assay

For the internalization assay, cells were washed twice with cold PBS pH 7.9 and subsequently cell surface molecules were biotinylated with 0.2 mg ml^−1^ sulfo-NHS-SS-biotin in the same buffer at 4 °C. The reaction was quenched 15 min later with 50 mM Tris and the cells were then incubated with prewarmed DMEM with 10% fetal calf serum for the indicated amount of time at 37 °C. Finally, cells were rapidly chilled again and surface exposed biotin was removed by incubating the cells for 3 × 7 min in 100 mM MESNA in MESNA buffer.

### Western blots

Samples were lysed in sample buffer (Novex, LC2676), boiled and run on precasted 4–20% Tris-Glycine gels (Invitrogen). The gels were then blotted using a semidry blotter (Bio-Rad), the membrane blocked in a PBS solution containing 4% dried skimmed milk powder, incubated with the appropriate primary antibodies, washed and incubated with HRP conjugated secondary antibodies (DAKO). The blots were then developed using Immobilon Western Chemiluminescent HRP Substrate (Millipore) on to Fuji Super RX X-ray films.

### Lipid extraction and thin layer chromatography)

Immortalized MEFs from wild type or caveolin 1−/− animals were loaded for 3 h at 37 °C with 5 μM BODIPY-Ganglioside M1 or 5 μM BODIPY-Lactosyl ceramide (both complexed with bovine serum albumin). The cells then were washed, lipids were extracted according to standard Bligh and Dyer method and separated on silica gel thin layer chromatography plates (Merck) with Chloroform/Methanol/Water (65:25:4). The fluorescent signal was then detected using Typhoon Trio instrument (Amersham Biosciences).

### Image processing and quantification

Images were processed and quantified in ImageJ. Where appropriate, images were subjected to Gaussian blur to remove noise and contrast was increased to remove background and facilitate display of three-colour overlays. To calculate the images of internalized CTB shown in Fig. 2b, both CTB images were normalized to have the same histogram of total pixel intensisties. Then the alexa647 image was subtracted from the alexa546 image and the resultant difference map was subjected to manual thresholding with reference to the raw data. This produced a binary image that could be used to isolate pixels in the caveolin-1-GFP image that are positive for internalized CTB. To calculate total endocytosis as in Fig. 8, mean fluorescence intensities from cell regions were background corrected using an empirically defined value derived from control cells labelled only at 4 °C. As there is inherent variability in both signal and background from cell to cell, this resulted in negative values in some instances. Data were normalized so that the mean of control cells=1. Endosome abundance was calculated by scoring the number of caveolar endosomes, defined as caveolin-1-GFP+internalized biotin-positive puncta, by eye. Co-localization values in Figs 4 and 7 were calculated using Image J. Two-channel raw images were acquired by confocal microscopy. The channels were separated, subjected to Gaussian blur with radius =0.7, and then contrast adjusted using the histogram of pixel intensities as shown. In the caveolin channel, the base of the histogram was used to set pixel intensity=0, maximal pixel intensity was not altered. In the biotin/streptavidin channel, which is used to generate a binary mask, pixel intensity=0 and maximal pixel intensity were both set to the base of the histogram of pixel intensities as shown. A logical ‘AND' operation was carried out to isolate those pixels in the caveolin channel that also are positive in the biotin/streptavidin binary mask. This image was combined with the original caveolin image in a two-colour overlay, and manually drawn regions of interest were used to calculate total pixel intensity in the caveolin channel, and total pixel intensity in the same channel from biotin/strptavidin-positive pixels.

### Statistical analysis

All data sets were analysed using Mann–Whitney non-parametric *t*-test, and statistical significance is displayed NS *P*>0.05, *P<0.05, **P<0.01, ***P<0.001, ****P<0.0001.

## Author contributions

E.S. performed the experiments, analysed the data and wrote the manuscript. V.B. performed the experiments. G.H. performed electron microscopy, C.G.H. generated cell lines, B.J.N. performed experiments, analysed the data, wrote the manuscript.

## Additional information

**How to cite this article:** Shvets, E. *et al*. Dynamic caveolae exclude bulk membrane proteins and are required for sorting of excess glycosphingolipids. *Nat. Commun.* 6:6867 doi: 10.1038/ncomms7867 (2015).

## Supplementary Material

Supplementary Figures Supplementary Figures 1-9

Supplementary Movie 1 Caveolin-1-GFP and cavin-1-mCherry co-localise in dynamic vesicles and static structures. TIR imaging. Genome edited NIH3T3 cell expressing caveolin-1-GFP and cavin-1-mCherry. Real elapsed time is shown top left. Movie acquired at 1Hz, plays back 15x real time. Region shown is approximately 5 microns across.

Supplementary Movie 2 Caveolin-1-GFP and cavin-1-mCherry in a motile structure with quantal size suggestive of individual caveola. TIR imaging. Movie acquired at 1Hz, plays back 15x

Supplementary Movie 3 Caveolin-1-GFP and cavin-1-mCherry in a motile structure with quantal size suggestive of cluster of caveolar quanta. TIR imaging. Movie acquired at 1Hz, plays back 15x

Supplementary Movie 4 Recovery of caveolin-1-GFP fluorescence in FRAP experiments is due to movement of vesicular structures. Movie covers a total of 5 minutes real time. Confocal imaging.

Supplementary Movie 5 Genome edited NIH3T3 cell expressing caveolin-1-GFP and cavin-1-mCherry, continuously labelled with CTB-alexa647 from 15 min prior to imaging. TIR images. Red arrows highlight motile structures containing all 3 markers. Movie acquired at 1Hz, plays back 15x real time.

Supplementary Movie 6 Genome edited NIH3T3 cell expressing caveolin-1-GFP and cavin-1-mCherry, continuously labelled with transferrin-alexa647 from 15 min prior to imaging. TIR images. Red arrows highlight motile structures containing caveolin 1 and cavin 1 but not transferrin. Movie acquired at 1Hz, plays back 15x real time.

Supplementary Movie 7 Genome edited NIH3T3 cell expressing caveolin-1-GFP and cavin-1-mCherry, continuously labelled with transferrin-alexa647 and CTB-alexa546 for 15 min prior to imaging. Greyscale panels are top left caveolin-1-GFP, top right CTB, bottom left transferrin. Red arrows highlight endosomal structure that moves in and out of the TIR field, and contains caveolin 1 and CTB but not transferrin. Movie acquired at 1Hz, plays back 15x real time.
