# Supplementary material for: Dynamic caveolae exclude bulk membrane proteins and are required for sorting of excess glycosphingolipids
Source: Nat Commun. 2015 Apr 21;6:6867. doi: 10.1038/ncomms7867 (PMC4410672; doi:10.1038/ncomms7867)
Supplement: Supplementary Figures — 1-9 [file ncomms7867-s1.pdf]

Supplementary Figure 1. In the absence of new protein translation, overexpressed caveolin-1-GFP is degraded faster than caveolin-1-GFP expressed from the endogenous *caveolin 1* locus

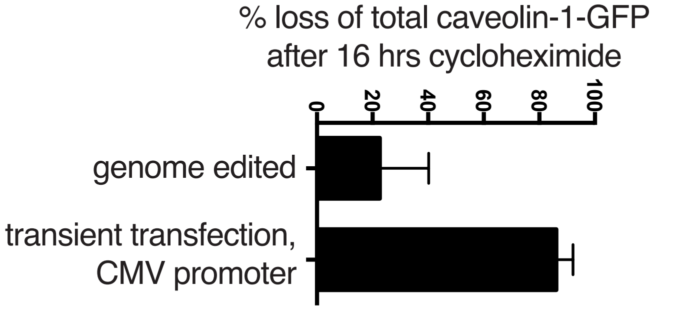

Supplementary Figure 1. In the absence of new protein translation, overexpressed caveolin-1-GFP is degraded faster than caveolin-1-GFP expressed from the endogenous *caveolin 1* locus. NIH3T3 cells expressing genome edited caveolin-1-GFP or NIH3T3 cells transiently expressing (24 hours) caveolin-1-GFP from a CMV promoter (pEGFP-N1, Clontech) were incubated for 16 hours in absence or presence of cyclohexamide (10 μg/ml). Cells were lysed and the level of caveolin-1-GFP, and endogenous caveolin 1 were detected by Western Blotting using anti-GFP and anti-caveolin-1 antibodies. Detection of actin with anti-actin antibody was used as loading control. The levels of caveolin-1-GFP or caveolin 1 were quantified using ImageJ software. Each treatment included two biological duplicates. For quantification, the level of caveolin-1-GFP in untreated cells was set to 100% and the relative percentage of caveolin-1-GFP in cyclohexamide-treated cells was calculated accordingly. Graph shows mean +/- SEM of duplicate samples from three independent experiments.

Supplementary Figure 2. Overexpressed caveolin-1-GFP is found in membrane structures devoid of cavin 1

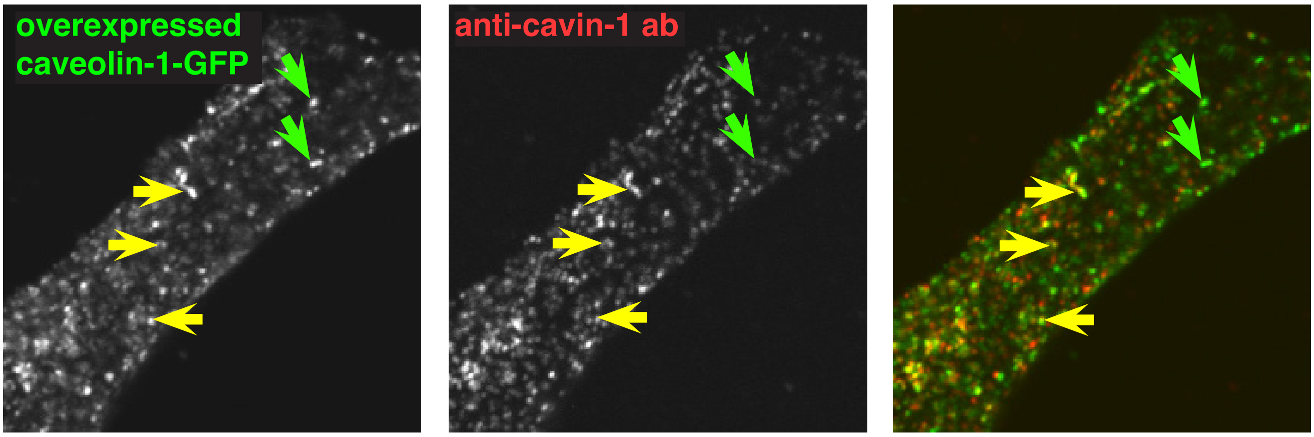

Supplementary Figure 2. Overexpressed caveolin-1-GFP is found in membrane structures devoid of cavin 1. Confocal image of NIH3T3 cells transiently transfected with caveolin-1-GFP (CMV promoter), fixed and stained with anti-cavin-1 antibody.

Supplementary Figure 3. Overexpression of caveolin-1-GFP increases the proportion of motile caveolin-1-positive vesicles

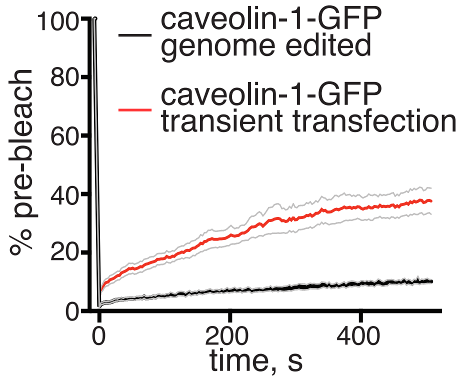

Supplementary Figure 3. Overexpression of caveolin-1-GFP increases the proportion of motile caveolin-1-positive vesicles. FRAP experiments were performed on cells expressing genome edited caveolin-1-GFP or overexpressed caveolin-1-GFP as described in the Methods section. Graphs represent mean+/-SEM of pooled data from three independent experiments. n=20 cells.

Supplementary Figure 4. Consecutivel labelling approach to identify intracellar, CTB-positive structures that also contain caveolin-1-GFP

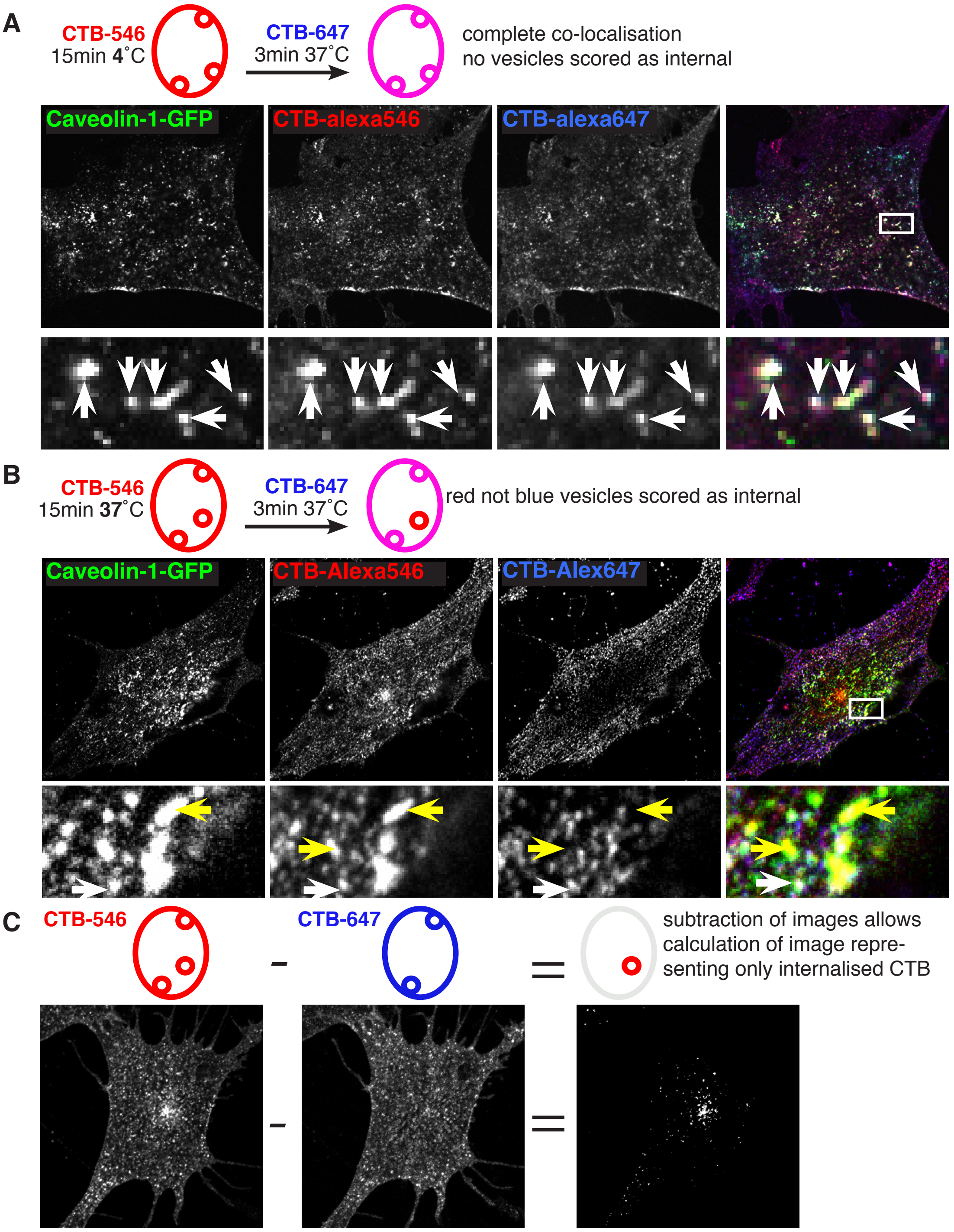

Supplementary Figure 4. Consecutive labelling approach to identify intracellular, CTB-positive structures that also contain caveolin-1-GFP. **A**. NIH3T3 cells expressing genome edited caveolin-1-GFP were labelled at 4˚C with CTB-alexa546 (1 μg/ml) for 15 min, then washed and labelled at 4C with CTB-alexa647 (1 μg/ml) for 15 min, washed again and transferred into 37˚C for short period of time (3 min). Fixed cells were analyzed using confocal microscope. White arrows indicate puncta containing all 3 fluors. The white box delineates region shown at high magnification in the lower panels. **B**. NIH3T3 cells expressing genome edited caveolin-1-GFP were labelled at 4˚C with CTB-alexa546 (1 μg/ml) for 15 min, then washed and incubated at 37˚C for 15min to allow internalisation, rapidly chilled, labelled at 4˚C with CTB-alexa647 (1 μg/ml) for 15 min, washed again and transferred into 37˚C for short period of time (3 min). Fixed cells were analyzed using confocal microscope. White arrows indicate caveolin-1-GFP puncta containing all 3 fluors, scored as extracellular, yellow arrows indicate puncta containing CTB-alexa647 but not CTB-alexa546, thereby scored as intracellular. The white box delineates region shown at high magnification in the lower panels. **C**. Illustrative example of consecutive labelling with different fluorescent CTB (first alexa546 and then alexa647 conjugates) allows image subtraction using ImageJ and thereby calculation of an image representing only internalised CTB structures.

Supplementary Figure 5. Controls to show that labelling of the cell surface with NHS-SS-biotin and treatment with a membrane-impermeant reducing agent provides a good assay for uptake from the plasma membrane

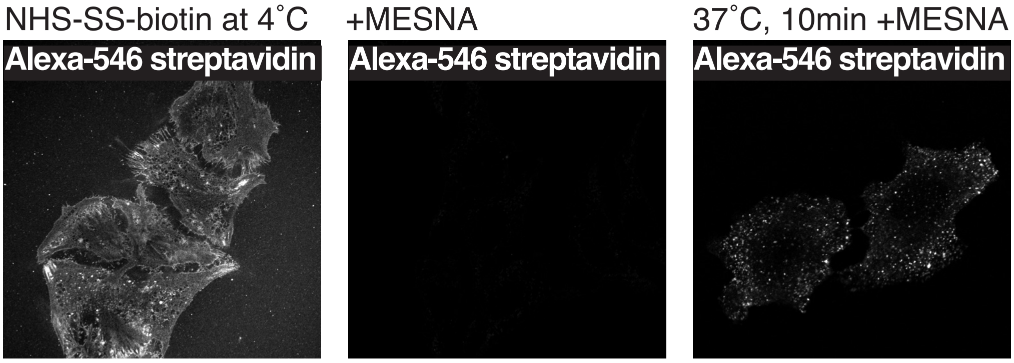

Supplementary Figure 5. Controls to show that labelling of the cell surface with NHS-SS-biotin and treatment with a membrane-impermeant reducing agent provides a good assay for uptake from the plasma membrane. Left image: Bulk cell surface molecules were reversibly biotinylated at 4˚C (to prevent internalisation) using Sulfo-NHS-SS-biotin as described in Materials and Methods Section. Biotinylated molecules were detected using alexa546 conjugated streptavidin following fixation and permeabilization. Middle image: Following biotinylation cells were kept on ice for 10min to prevent internalisation and treated with cell-impermeable reducing agent MESNA to reduce biotin from cell-surface molecules. Biotinylated molecules were detected using alexa546 conjugated streptavidin following fixation and permeabilization. Note that this treatment completely abolished any signal coming from biotinylated molecules indicating complete removal of biotin when no internalisation occurred. Right image: Following biotinylation cells were transferred to 37˚C to induce internalisation and then treated with cell-impermeable reducing agent MESNa to detach biotin from cell-surface molecules. Biotinylated molecules were detected using alexa546 conjugated streptavidin following fixation and permeabilization. Note that now numerous punctate structures protected from reducing agent were detected in the cells, thus representing pool of internalised molecules in endosomes.

Supplementary Figure 6. Caveolin-1-GFP and cavin-1-mCherry expressed by genome editing co-localise in caveolar endosomes

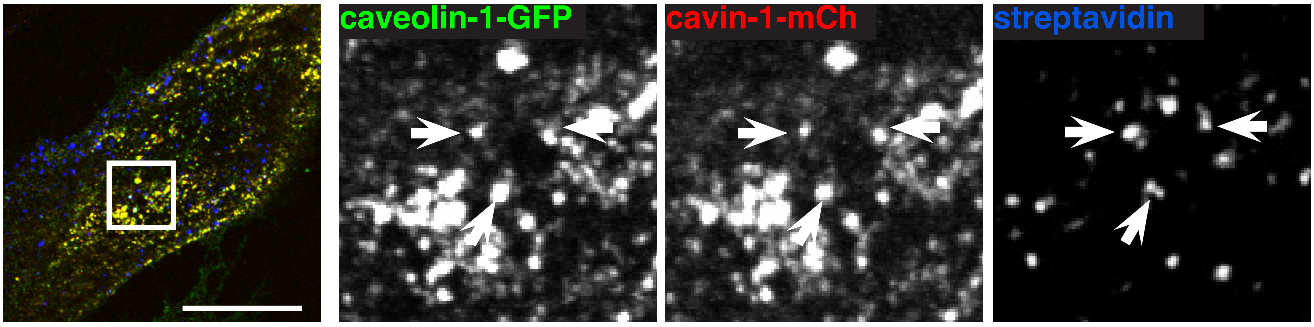

Supplementary Figure 6. Caveolin-1-GFP and cavin-1-mCherry expressed by genome editing co-localise in caveolar endosomes. Confocal image of internalised sulfo-NHS-SS-Biotin labelling surface free amine groups, after 15min uptake and removal of extracellular biotin using the membrane impermeant reducing agent MESNA. Biotin was detected with fluorescent streptavidin. Arrows indicate co-localisation between caveolin-1-GFP, cavin-1-mCherry and biotin in caveolar endosomes. Bar is 10μ, the white box delineates the region shown at higher magnification in the 3 right-hand panels.

Supplementary Figure 7. Binding and initial endocytosis of GM1 and lactosyl ceramide is not detectably altered in *caveolin 1-/-* cells

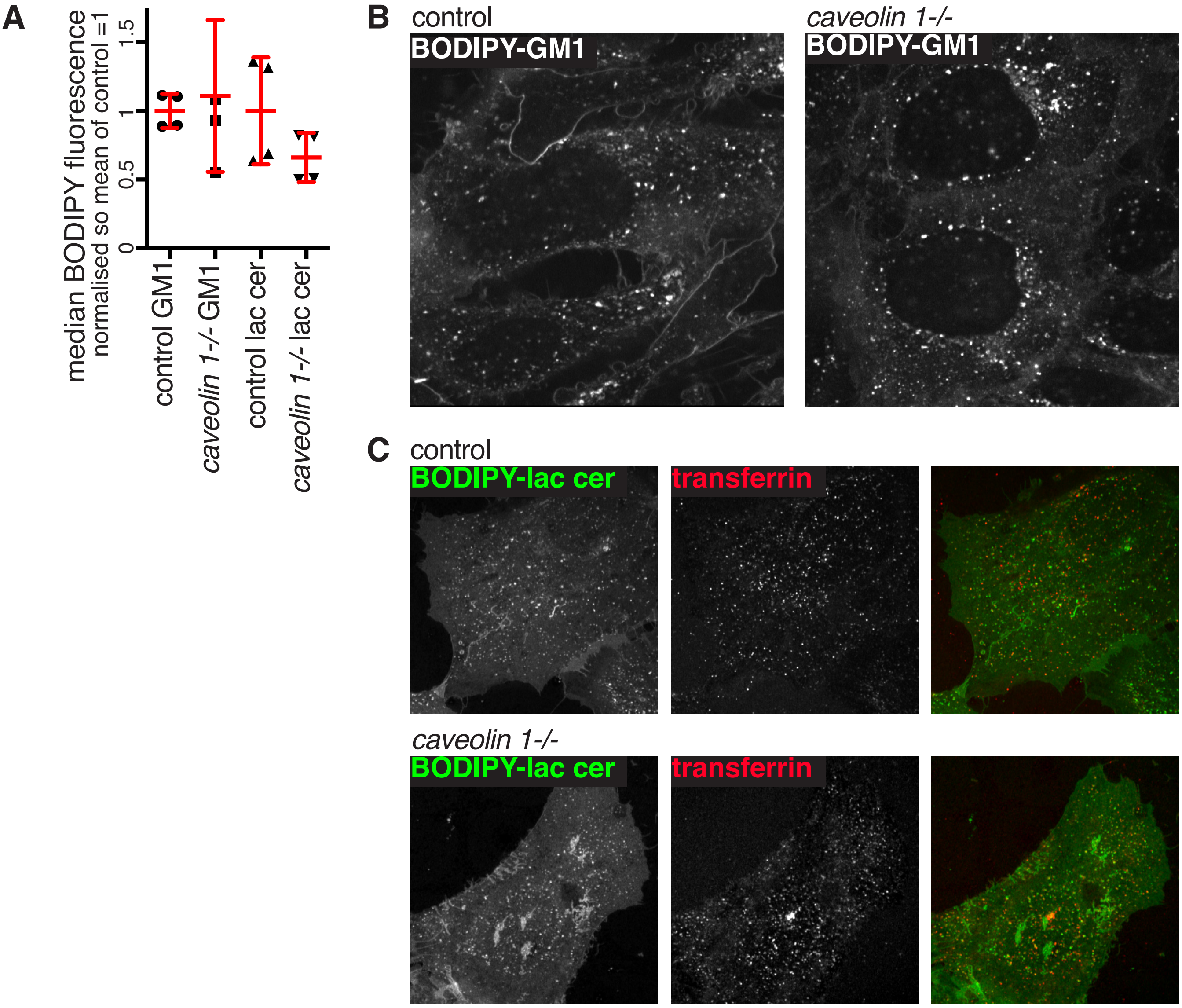

Supplementary Figure 7. Binding and initial endocytosis of GM1 and Lactosyl Ceramide is not detectably altered in *caveolin 1-/-* cells. **A**. Quantification of BODIPY-GM1 or BODIPY-LacCer amounts using flow cytometry. Live immortalised MEFs from congenic control or *caveolin-1* -/- animals were labelled for 1 hour at 4˚C with 5µM of BODIPY-lactosyl ceramide or BODIPY-GM1, washed, re-suspended and the amount of lipids associated with cells were measured using flow cytometry. Graph represents mean +/- SEM of two different clones in two separate experiments. Note that no significant difference is observed between control and *caveolin 1* -/- cells at this time point. **B**. Confocal images of live immortalised MEFs from control or *caveolin 1* -/- animals incubated for 15min at 37˚C with 5µM BODIPY-GM1. **C**. Confocal images of live immortalized MEFs from control and *caveolin 1* -/- animals incubated for 15min at 37˚C with 5µM BODIPY- Lactosyl ceramide and 0.2 mg/ml of alexa647-Transferrin. Note that no significant difference is observed between control and caveolin 1-/- cells at this time point.

Supplementary Figure 8. Thin layer chromatography reveals that metabolism of BODIPY-lactosyl ceramide and BODIPY-GM1 is not altered in *caveolin 1* knockout cells

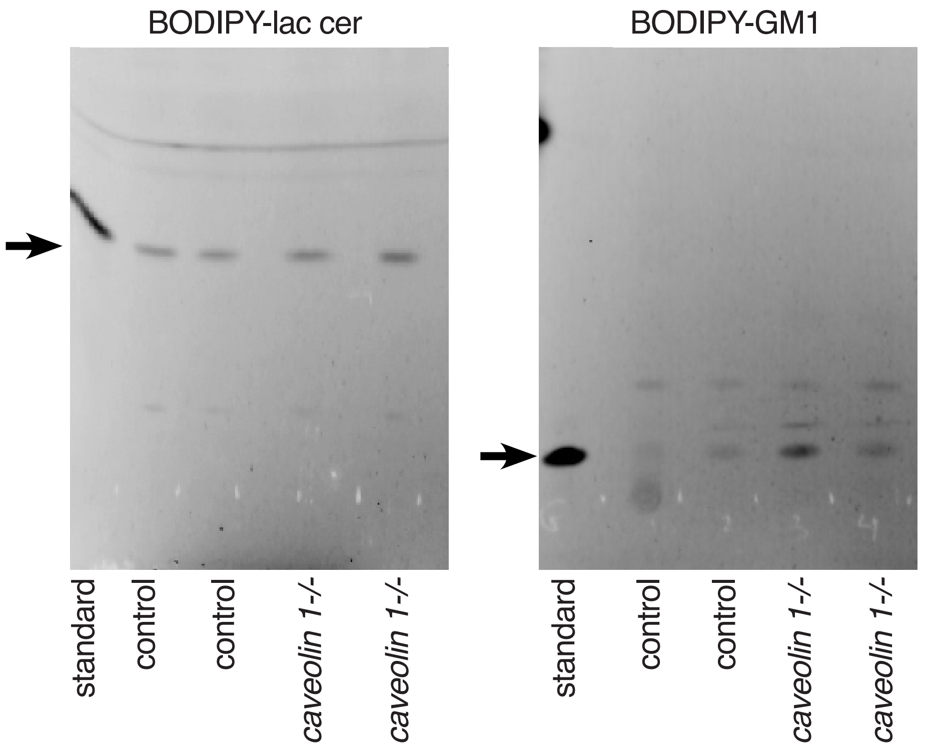

Supplementary Figure 8. Thin layer chromatography reveals that metabolism of BODIPY-lactosyl ceramide and BODIPY-GM1 is not altered in caveolin 1 knockout cells. Live immortalised MEFs from congenic control or caveolin 1 -/- animals (two different clones from each genotype) were labeled for 3 hours at 37˚C with 5µM of BODIPY-lactosyl ceramide or BODIPY-GM1. The cells then were washed and lipids were analysed as described in Methods section. Arrows point on the position on the chromatogram of standard samples of BODIPY-lactosyl ceramide or BODIPY-GM1.

Supplementary Figure 9. Magic red cathepsin assay suggests that lysosomal function is not impaired in *caveolin 1* knockout cells

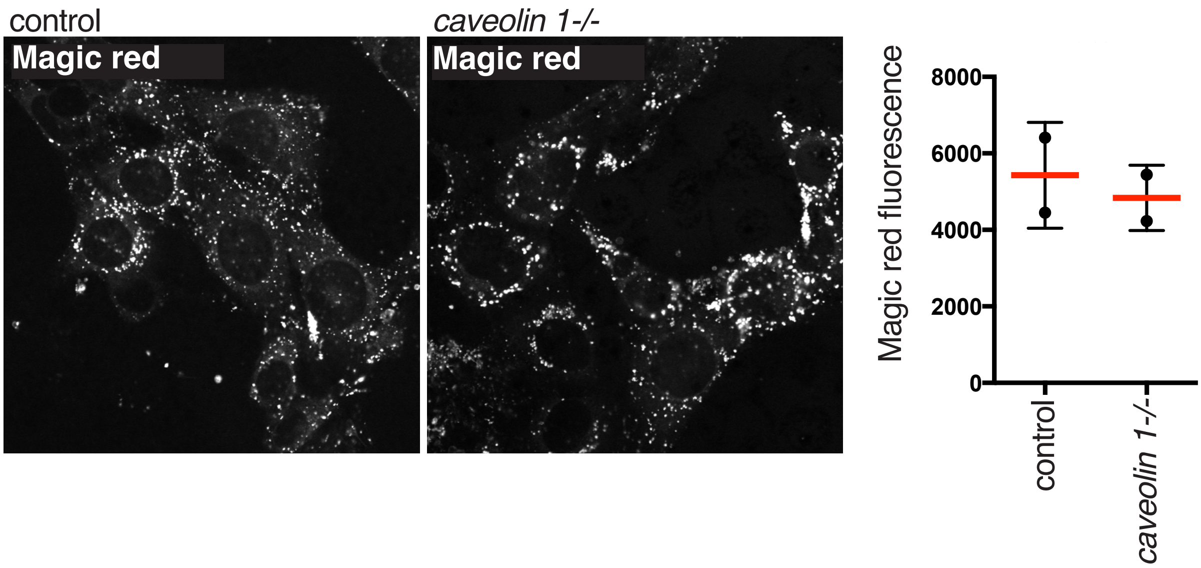

Supplementary Figure 9. Magic red cathepsin assay suggests that lysosomal function is not impaired in *caveolin 1* knockout cells. Left: Confocal images of live immortalised MEFs from control or *caveolin 1* -/- animals loaded for 3 hours at 37˚C with non-fluorescent GM1. Lysosomal activity was detected by incubating cells with Magic Red Cathepsin L kit, according to manufacturer instructions. Right: Quantification of lysosomal activity using flow cytometry analysis. Immortalised MEFs from control or *caveolin 1* -/- animals were loaded for 3 hours at 37˚C with non-flourescent GM1. Cells were then incubated with Magic Red according to manufacturer instructions and the fluorescence was quantified by flow cytometry. Data from two control and two knockout clones analysed in a single representative experiment are shown.
